# Uptake of oral fluid-based HIV self-testing among men who have sex with men and transgender women in Thailand

**DOI:** 10.1371/journal.pone.0256094

**Published:** 2021-08-16

**Authors:** Philippe Girault, Christina Misa Wong, Siroat Jittjang, Kangwan Fongkaew, Michael M. Cassell, Cheewanan Lertpiriyasuwat, Ravipa Vannakit, Matthew Avery, Danai Linjongrat, Saman Sumalu, Somchai Phromsombat, Nuttakrit Mainoy, Pongpeera Patpeerapong, Phonpiphat Potasin, Pornpichit Brutrat, Stephen Mills, Nittaya Phanuphak, Praphan Phanuphak

**Affiliations:** 1 FHI 360, Asia Pacific Regional Office, Bangkok, Thailand; 2 FHI 360, Behavioral, Epidemiological and Clinical Sciences, Durham, North Carolina, United States of America; 3 Burapha University, Chonburi, Thailand; 4 USAID Regional Development Mission for Asia, Bangkok, Thailand; 5 Thai Ministry of Public Health, Nonthaburi, Thailand; 6 Rainbow Sky Association of Thailand, Bangkok, Thailand; 7 Service Workers in Group Foundation, Bangkok, Thailand; 8 The Poz Home Center Foundation, Bangkok, Thailand; 9 Caremat, Chiang Mai, Thailand; 10 Mplus Foundation, Chiang Mai, Thailand; 11 Sister, Pattaya, Thailand; 12 Service Workers in Group Foundation, Pattaya, Thailand; 13 Institute of HIV Research and Innovation, Bangkok, Thailand; 14 Thai Red Cross AIDS Research Centre, PREVENTION, Bangkok, Thailand; University of Toronto, CANADA

## Abstract

**Background:**

Suboptimal uptake of HIV testing remains a primary bottleneck to HIV prevention and treatment for men who have sex with men (MSM) and transgender women (TGW) in Thailand. The World Health Organization has recommended HIV self-testing (HIVST) as an additional strategic HIV service. However, HIVST has not been fully endorsed and implemented in many countries in Southeast Asia. The aim of this study was to assess the uptake of oral fluid-based HIVST in MSM and TGW populations in Thailand.

**Methods:**

During 2017 and 2018, we conducted a cross-sectional study using convenience sampling to enroll 2,524 participants from three major urban areas. Participants were recruited during outreach and online activities and were offered unassisted or assisted HIVST, or referral to HIV testing services. A descriptive analysis was performed for summarizing data.

**Results:**

A total of 2,502 participants (1,422 MSM and 1,082 TGW) were included in the analysis with about one-third (36.1%) of them being first-time testers. Among all participants enrolled in the study, a total of 2,486 participants (99.3%) selected HIVST versus referral to HIV testing services. Of those who selected HIVST, 2,095 (84.3%) opted for assisted HIVST while the rest opted for unassisted HIVST: 1,148 of 1,411 MSM (81.4%) and 947 of 1,075 TGW (88.1%) selected assisted HIVST. While no serious adverse events were reported during the study, we found that among 179 participants who needed a confirmatory test and were referred to HIV testing services, 108 (60.3.4%) accessed these later services.

**Conclusions:**

This study demonstrated a high uptake of oral fluid-based HIVST among MSM and TGW populations in Thailand and that HIVST could be scaled up through the national epidemic control program. However, a better understanding of HIV testing-seeking behavior and innovative follow-up solutions are needed to improve and monitor linkages to services for people who undertake HIVST.

## Introduction

Men who have sex with men (MSM) and transgender women (TGW) are disproportionately affected by HIV globally [1, 2], including in Thailand [3, 4], but face barriers to access to HIV prevention and treatment services. Antiretroviral medications have been proven to prevent HIV-related illness and death as well as sexual transmission of the virus, and HIV pre-exposure prophylaxis (PrEP), correct and consistent condom use, and several other effective resources exist to prevent HIV infection. HIV testing is a key entry point into the full cascade of services [5–7] but, unfortunately, uptake of HIV testing services among MSM and TGW has been low across regions of the world. This remains the case despite structural improvements toward differentiated HIV testing modalities (e.g., rapid testing and task shifting to nonprofessional service providers); demand generation activities (peer education, promotional campaigns); and, in many settings, increased availability of HIV treatment [1, 2, 8].

Since the World Health Organization (WHO) published its guidelines on HIV self-testing (HIVST) and partner notification with recommendations for global scale-up in 2016 [9], there has been increasing interest globally in the reliability, discreetness, and convenience of HIVST. However, some governments and practitioners remain skeptical for various reasons—including concerns about cost-effectiveness and a resistance to shift HIV testing activities to self-care and lay providers—and have not yet embraced HIVST as a standard public health practice in many countries, including Thailand.

Differentiating HIV testing modalities is critical to achieving global HIV epidemic control by 2030. Diversifying HIV testing options can help increase access to HIV diagnosis, treatment, and prevention services for those with different needs and life circumstances [10, 11]. WHO recommends that “HIV self-testing should be offered as an additional approach for HIV testing, especially to reach first-time testers, people with unknown HIV status, and those with ongoing risk” [9]. Systematic reviews are contributing to a growing global evidence base for the inclusion of HIVST in national strategies. Findings suggest that HIVST is reaching priority populations and is preferred to facility-based testing because of its convenience and confidentiality [12]. They also suggest that oral fluid-based HIVST is preferred over blood-based testing [13]; self-testers can reliably and accurately conduct HIVST [14]; oral fluid-based HIVST is highly acceptable and preferred by key populations, particularly MSM [15]; and HIVST is associated with increased uptake and frequency of testing in randomized controlled trials [16]. Global studies have also shown that few to no serious adverse effects have been reported as a result of HIVST [17–21].

The Linkages across the Continuum of HIV Services for Key Populations Affected by HIV (LINKAGES) project implemented in Thailand from 2015 to 2020 focused on the development of effective and sustainable systems to reach, test, treat, and retain MSM and TGW in HIV prevention, treatment, care, and support services. This project used the HIV cascade of services as its overall strategic framework with the goal of increasing the number of HIV-positive individuals who know their status and are receiving antiretroviral therapy (ART). However, suboptimal uptake of HIV testing services remains a primary bottleneck within this cascade toward fully implementing the Royal Thai Government’s Universal HIV Testing and Treatment policy. According to national data in 2016, HIV testing coverage among MSM in Thailand was estimated to be 29% [22] while LINKAGES monitoring data for the year 2016 indicated that among MSM and TGW reached by community-based organizations (CBOs) working in three urban centers, 44% of them had an HIV test and received their results (M. Avery, personal communication, May 15, 2021). Until recently, Thailand’s HIV testing policy and regulatory frameworks did not include HIVST as an option for HIV testing.

In 2016, the LINKAGES project and key partners proposed a study to explore the uptake of fluid-based HIVST (OraQuick^®^) versus routine referral to facility for blood-based HIV testing among MSM and TGW reached through community-based outreach activities in Thailand.

## Methods

### Study design and setting

Between April 2017 and December 2018, the LINKAGES project collaborated with the Thai Ministry of Public Health, the Thai Red Cross AIDS Research Centre, and six key population-led CBOs to conduct a cross-sectional study at seven study sites in the metropolitan areas of Bangkok, Chiang Mai, and Pattaya cities.

### Study population

The study was conducted among Thai MSM and TGW reached through community-based outreach activities. Eligibility criteria for enrollment included being a Thai citizen age 15 years or older, having self-reported anal or oral sex with at least one male sexual partner in the past 12 months, being willing to provide contact information (e.g., telephone number or social media username) and be contacted by study staff to allow follow-up, and having reported recent HIV-negative status or unknown HIV status. People who had already initiated ART or had taken pre- or post-exposure prophylaxis in the past three months were not eligible to avoid the risk of false-negative HIVST results, although rare, among individuals taking HIV antiretroviral drugs [23].

### Sampling frame

Convenience sampling was used to recruit individuals into the study during routine physical and virtual outreach activities implemented by the CBOs. Outreach teams at the seven study sites approached and recruited potentially eligible individuals during their routine activities. All individuals reached during off-line and online outreach activities who met the eligibility criteria were approached to participate in the study. A total of 1,432 MSM and 1,092 TGW were enrolled in the study.

### HIV testing

This study used the OraQuick^®^ rapid HIV-1/2 antibody test, a qualitative, in vitro immunoassay for detecting HIV types 1 and 2 (HIV-1/2) in human oral fluid, whole blood, serum, and plasma. The OraQuick^®^ HIV Self-Test, which is manufactured in Thailand, was accepted for the WHO list of prequalified in vitro diagnostics in 2016 with a reported sensitivity of 99.1% (95% CI: 94.8%–100%) and specificity of 100% (95% CI: 99.0%–100%) [24]. Since HIVST was not authorized in Thailand and the OraQuick^®^ HIV Self-Test kit was not registered by the Food and Drug Administration of the Thai Ministry of Public Health, the study secured Thai FDA approval to procure and use these test kits under research activities.

Participants who declined HIVST, had a reactive or invalid HIVST result, or were not able to interpret the HIVST result, were referred to community- or facility-based HIV testing services for confirmatory testing following the national HIV diagnostic algorithm including the following HIV tests: Alere Determine HIV-1/2 (third generation), Colloidal Gold Device, and SD Bioline HIV-1/2. Participants confirmed HIV positive were referred for ART initiation and HIV services at CBOs and collaborating public and private facilities.

### Study procedures

Eligible MSM and TGW clients were given a brief explanation of the study and were offered three HIV testing options: unassisted HIVST, assisted HIVST, or routine referral to a facility for blood-based HIV testing.

Unassisted HIVST was defined as provision of an HIVST kit that included detailed instructions for conducting the self-test and interpreting the results. The unassisted HIVST kit provided by the study team included: (1) manufacturer’s insert with pictorials and text; (2) a unique identifier code to access secure pages on the Thai-language study website with a step-by-step video on HIVST, an online questionnaire, and a place to report test results; and (3) a referral card with contact information (e.g., the telephone number or social media username) of trained outreach workers to provide support and assistance and of selected health facilities for confirmatory HIV testing as needed. Assisted HIVST was defined as provision of an oral fluid-based HIVST kit by a trained outreach worker along with oral instructions before and during the procedure, and, when requested, assistance in conducting the test and/or interpreting the result.

Participants who opted for unassisted HIVST could receive a self-test kit directly from an outreach worker in the community, at a CBO facility, or via express mail service. Those who selected assisted HIVST were invited to conduct the test at a private venue chosen by the participants, near the location where they were recruited. OraQuick^®^ kits were offered to all participants free of charge.

Before collecting data, informed consent was administered either face-to-face or—for clients recruited via social media—via a phone call. Collaborating CBOs’ staff systematically followed up with participants via phone or social messaging apps (privately) to support linkage to relevant services, monitor adverse events within 48 hours after unassisted or assisted HIVST, and, for those who opted for unassisted HIVST, to document that the test had been received and conducted. All HIV testing and treatment services were free of charge following standard procedures under the national AIDS program.

### Data collection tools

The structured questionnaire (S1 and S2 Texts) included 10 different sections: (1) sociodemographic characteristics; (2) sexual behavior in the past three months; (3) health-seeking behavior for HIV testing; (4) experience of stigma and discrimination; (5) HIV knowledge and awareness, experience of HIV self-testing; (6) perceived concerns and benefits of HIV testing; (7) intention, self-efficacy, and willingness to pay; (8) substance use; (9) exposure to HIV interventions in the community; and (10) medication history. For participants who selected unassisted HIVST, two other sections were added: (11) self-reported HIVST testing result and (12) experience with oral fluid-based HIVST. For participants who opted for assisted HIVST, this questionnaire (sections 1–10) was self-administered or administered by an outreach worker if the participants declined self-administered method, using the CommCare^™^ (Dimagi, Inc., USA) application. Participants who opted for unassisted HIVST were invited to complete the online questionnaire (sections 1–12) through the study website. Prior to submitting the protocol for ethical review, the research team pretested the questionnaire with MSM and TGW staff at participating CBOs to assess the comprehension and acceptability of the wording, the logic and flow of the questions, and the length of the interview. A first group of 37 MSM and TGW were involved in the initial pretesting. After addressing comments from the first group and adjusting the questionnaire, a second group of 52 MSM and TGW were asked to pretest the final version. Furthermore, the CommCare application and survey platform were tested with data created by the research team to ensure these systems operated correctly and smoothly.

A structured case report form for adverse events was administered through an audio phone call and completed by the counselors of the community-based HIV testing services. The counselors contacted each participant 48 hours after completing the HIVST procedure for those who opted for assisted HIVST, or after reporting HIVST result on the dedicated website for those who selected unassisted HIVST. The form included questions on major signs or behaviors related to emotional and cognitive stress and suicidal ideation, attempts of suicide, nonsuicidal self-injury, alcohol or drug binging, and experience of social harm. The participants were asked to report the occurrence of these signs and behaviors in the past 12 months prior to the HIVST procedure and after obtaining the HIVST results.

### Data management and analysis

All electronic and hard copy audio files, questionnaires, case report forms, expanded notes, transcripts, and forms were stored securely at each of the study sites at the drop-in centers, without any personal identifiers.

Data from CommCare and the study’s website were exported into Excel 2016 files. Using Stata 15 (College Station, TX: StataCorp LLC; 2017), these Excel files were imported and then appended into a single matrix. Data from case reporting forms (e.g., test results and adverse events) were also converted into Stata files and merged with the single matrix using the unique study identification codes of the participants. The final dataset was then created and cleaned to correct, as much as possible, discrepancies and to minimize missing values by reviewing the participants’ study files stored at each study site.

During the preliminary analyses, continuous variables such as age in years and price to pay for HIVST kit were collapsed into categorical variables. New variables were created such as “first-time vs. repeat testers” using responses on HIV testing history and linkages to facility for HIV confirmatory testing and treatment initiation using data from respective case reporting forms. The occurrence of each adverse event related to HIVST was coded as “true” when the respondent reported the manifestation of specific signs, behaviors, or social harm after undergoing HIVST but not in the past 12 months. Binary variables were then constructed for each category of adverse event.

Descriptive analysis (frequency distributions and measures of central tendency) was performed with Stata 15. For each population (MSM and TGW), frequency of participants and proportions were calculated to assess the uptake of HIVST as the primary outcome of the study. Similar analysis was performed to describe participants’ characteristics, history of HIV testing and exposure to interventions, outcomes of HIV testing, linkages to services, and intention to use HIVST in the future. Measures of central tendency were done for age in years and price to pay for HIVST. Cross-tabulations were also performed to identify patterns between mode of recruitment and testing modalities, and testing modalities and outcomes of HIV testing and linkages to services.

### Ethical considerations

The protocol and tools were approved by Chulalongkorn University’s Research Ethics Review Committee for Research Involving Human Subjects in Thailand and FHI 360’s Protection of Human Subjects Committee in the United States of America. Participants received a compensation of 500 Thai baht (about US$15.50 in 2017) for time and travel expenses for taking part in the study. All participants confirmed HIV positive with the national HIV testing algorithm were referred to clinical services for ART initiation at no cost.

## Results

### Enrollment and exclusion

A total of 2,524 participants (1,432 MSM and 1,092 TGW) were enrolled in the study to assess the uptake of oral fluid-based HIVST. However, 20 participants were excluded from the analysis: five participants (two MSM and three TGW) received the kit for unassisted HIVST but did not enter their result and complete the online questionnaire despite follow-up contacts by phone; five (four MSM and one TGW) were discovered to be known HIV positive and receiving ART; and one MSM was excluded from the database—after receiving recommendations from institutional review boards—for suspicion of coercion. An additional nine participants (three MSM and six TGW) were duplicate cases across research sites—after confirming with the concerned participants and research sites, only the observation from their first enrollment was kept. After exclusion, the total sample size used for the analysis was 2,504 participants (1,422 MSM and 1,082 TGW). A total of 1,966 (78.5%) participants were enrolled through face-to-face contact (1,092 MSM, 76.8%; 874 TGW, 80.8%) while 538 (21.5%) were enrolled through social media (330 MSM, 23.2%; 208 TGW, 19.2%).

### Key sociodemographic characteristics of participants

Table 1 shows the key sociodemographic characteristics of 1,422 MSM and 1,082 TGW enrolled in the study. The mean age of MSM was 26.5 years (standard deviation [*SD*] = 8.1) with a range from 15 to 71 (median = 24) while the mean age for TGW was 25.7 years (*SD* = 7.7) with a range from 15 to 57 (median = 23). About one-half of MSM (54.3%) and TGW (55.9%) were between ages 15 and 24, with 15.3% of MSM and 18.9% of TGW aged less than 20. Most of them reported being single (87.6% MSM, 89.7% TGW) and having completed high school or university level education (74.4% MSM, 72.8% TGW). Around one-third reported being a student (30.7% MSM, 35.0% TGW) and living alone (32.1% MSM, 33.8% TGW).

**Table 1:** Key sociodemographic characteristics of participants.

	MSM		TGW		Total	
	*n*	*%*	*n*	*%*	*n*	*%*
**Total participants (N)**	1,422	100%	1,082	100%	2,504	100%
**City**						
*Bangkok*	646	45.4%	502	46.4%	1,148	45.8%
*Chiang Mai*	500	35.2%	400	37.0%	900	35.9%
*Pattaya*	276	19.4%	180	16.6%	456	18.2%
**Age (years)**						
*15–19*	217	15.3%	204	18.9%	421	16.8%
*20–24*	555	39.0%	400	37.0%	955	38.1%
*25–29*	248	17.4%	213	19.7%	461	18.4%
*≧ 30*	402	28.3%	265	24.5%	667	26.6%
**Highest level of education completed**						
*No schooling*	8	0.6%	8	0.7%	16	0.6%
*Primary*	93	6.5%	45	4.2%	138	5.5%
*Secondary*	209	14.7%	192	17.7%	401	16.0%
*High-school*	617	43.4%	564	52.1%	1,181	47.2%
*University*	441	31.0%	224	20.7%	665	26.6%
*No answer*	54	3.8%	49	4.5%	103	4.1%
**Marital status**						
*Married*	37	2.6%	2	0.2%	39	1.6%
*Divorced*	4	0.3%	2	0.2%	6	0.2%
*Widowed*	3	0.2%	2	0.2%	5	0.2%
*Single*	1,245	87.6%	971	89.7%	2,216	88.5%
*No answer*	133	9.4%	105	9.7%	238	9.5%
**Occupation**						
*Unemployed*	80	5.6%	64	5.9%	144	5.8%
*Student*	436	30.7%	379	35.0%	815	32.5%
*Civil servant*	35	2.5%	6	0.6%	41	1.6%
*Self-employee*	151	10.6%	122	11.3%	273	10.9%
*Employee*	487	34.2%	358	33.1%	845	33.7%
*Other*	170	12.0%	109	10.1%	279	11.1%
*No answer*	63	4.4%	44	4.1%	107	4.3%
**Living with**						
*Alone*	456	32.1%	366	33.8%	822	32.8%
*Friends*	292	20.5%	256	23.7%	548	21.9%
*Family*	427	30.0%	330	30.5%	757	30.2%
*Female partner*	48	3.4%	1	0.1%	49	2.0%
*Male partner*	158	11.1%	106	9.8%	264	10.5%
*No answer*	41	2.9%	23	2.1%	64	2.6%

### Behavioral characteristics, history of HIV testing and exposure to interventions

Approximately two-fifths of participants reported having found their last sexual male partner through social media (39.6% MSM, 43.1% TGW). Use of a condom at last anal sex with casual partner was reported by a majority (80.4% MSM, 78.3% TGW). A minority of MSM (3.7%) and TGW (2.6%) acknowledged they had ever injected a recreational drug, while 13.0% MSM and 7.8% TGW reported they had ever swallowed, snorted, or smoked recreational drugs (Table 2).

**Table 2:** Key behavioral characteristics of participants.

	MSM		TGW		Total	
	*n*	*%*	*n*	*%*	*n*	*%*
**Total participants (N)**	1,422	100	1,082	100	2,504	100
**How met last sexual male partner**						
Social media	563	39.6%	466	43.1%	1,029	41.1%
Friend referral	124	8.7%	127	11.7%	251	10.0%
Private party	27	1.9%	44	4.1%	71	2.8%
Private sex party	2	0.1%	3	0.3%	5	0.2%
Bar/discotheque	186	13.1%	137	12.7%	323	12.9%
Sauna/massage parlor	119	8.4%	25	2.3%	144	5.8%
Other	66	4.6%	61	5.6%	127	5.1%
No answer	335	23.6%	219	20.2%	554	22.1%
**Role during anal sex in past 3 months**						
Always insertive	543	38.2%	25	2.3%	568	22.7%
Always receptive	290	20.4%	773	71.4%	1,063	42.5%
Both roles	396	27.8%	156	14.4%	552	22.0%
No anal sex in past 3 months	103	7.2%	65	6.0%	168	6.7%
No answer	90	6.3%	63	5.8%	153	6.1%
**Condom use at last anal sex with casual male partner** *						
No	188	19.6%	165	21.7%	353	20.5%
Yes	770	80.4%	596	78.3%	1,366	79.5%
**Ever injected recreational drugs**						
Never	1,294	91.0%	980	90.6%	2,274	90.8%
Past 3 months	14	1.0%	15	1.4%	29	1.2%
Between 3–12 months	14	1.0%	4	0.4%	18	0.7%
> 1 year	24	1.7%	9	0.8%	33	1.3%
No answer	76	5.3%	74	6.8%	150	6.0%
**Ever swallowed/snorted/smoked recreational drugs**						
Never	1,147	80.7%	921	85.1%	2,068	82.6%
Past 3 months	88	6.2%	39	3.6%	127	5.1%
Between 3–12 months	39	2.7%	22	2.0%	61	2.4%
> 1 year	59	4.1%	24	2.2%	83	3.3%
No answer	89	6.3%	76	7.0%	165	6.6%

* Among participants who reported casual partners: MSM, N = 1,107; TGW, N = 876.

Around one-third of MSM (34.5%) and TGW (38.3%) said they had never been tested for HIV and were considered “first-time testers” under this study (Table 3). Among participants who had ever been tested for HIV, 33.4% MSM and 37.2% TGW received their most recent test in fixed or mobile services managed by MSM and TGW CBOs. Furthermore, a majority of MSM (74.6%) and TGW (74.5%) reported having not been exposed in the past 12 months to HIV interventions tailored to MSM and TGW (Table 3).

**Table 3:** History of HIV testing and exposure to interventions.

	MSM		TGW		Total	
	*n*	*%*	*n*	*%*	*n*	*%*
**Total participants (N)**	1,422	100%	1,082	100%	2,504	100%
**Last HIV test done**						
*Never tested (first-time tester)*	491	34.5%	414	38.3%	905	36.1%
*In past 3 months*	104	7.3%	67	6.2%	171	6.8%
*Between last 3 to 6 months*	201	14.1%	153	14.1%	354	14.1%
*Between last 6 to 12 months*	193	13.6%	186	17.2%	379	15.1%
*More than 12 months*	351	24.7%	210	19.4%	561	22.4%
No answer	82	5.8%	52	4.8%	134	5.4%
**Place of last HIV test** *						
*Community-based clinic* ^†^	159	18.7%	150	24.4%	309	44.5%
*Private health facility*	144	17.0%	85	13.8%	229	32.9%
*Anonymous clinic of TRC*	100	11.8%	57	9.3%	157	22.6%
*Governmental health facility*	187	22.0%	135	21.9%	322	41.8%
*Mobile services* ^†^	125	14.7%	79	12.8%	204	26.5%
*Research / surveillance*	33	3.9%	30	4.9%	63	8.2%
*Other*	7	0.8%	11	1.8%	18	2.3%
No answer	94	11.1%	69	11.2%	163	21.2%
**Exposed to tailored HIV interventions (past 12 months)**						
*No*	1,061	74.6%	806	74.5%	1,867	74.6%
*Yes*	272	19.1%	196	18.1%	468	18.7%
*No answer*	89	6.3%	80	7.4%	169	6.7%

*Among those who reported ever been tested: MSM, N = 849; TGW, N = 616.

^†^Managed by MSM and TGW community-based organizations.

TRC: Thai Red Cross.

### Uptake of HIV self-testing

Among all participants enrolled in the study, a total of 2,486 participants (99.3%) selected HIVST versus referral to HIV testing services. As shown in Table 4, most MSM and TGW opted for assisted versus unassisted HIVST or referral to a facility. Among MSM (N = 1,422), 1,148 (80.7%) elected assisted HIVST versus other HIV testing modalities offered under this study. Of the MSM who opted for unassisted HIVST (N = 260), 190 (72.2%) preferred receiving the HIVST kit through express mail service versus face-to-face contact with a community-based staff member either in the community or at the CBO facility. Among TGW enrolled in the study (N = 1,082), 947 (87.5%) opted for assisted HIVST. Of the TGW who selected unassisted HIVST (N = 128), 60 (46.9%) chose the express mail service distribution option and 50 (39.1%) received the kit from a peer outreach worker in the community (Table 4).

**Table 4:** Uptake of HIVST by population.

	MSM		TGW		Total	
	*n*	*%*	*n*	*%*	*n*	*%*
**Total participants (N)**	1,422	100%	1,082	100%	2,504	100%
**Last HIV test done**						
*Never tested (first-time tester)*	491	34.5%	414	38.3%	905	36.1%
*In past 3 months*	104	7.3%	67	6.2%	171	6.8%
*Between last 3 to 6 months*	201	14.1%	153	14.1%	354	14.1%
*Between last 6 to 12 months*	193	13.6%	186	17.2%	379	15.1%
*More than 12 months*	351	24.7%	210	19.4%	561	22.4%
No answer	82	5.8%	52	4.8%	134	5.4%
**Place of last HIV test** *						
*Community-based clinic* ^†^	159	18.7%	150	24.4%	309	44.5%
*Private health facility*	144	17.0%	85	13.8%	229	32.9%
*Anonymous clinic of TRC*	100	11.8%	57	9.3%	157	22.6%
*Governmental health facility*	187	22.0%	135	21.9%	322	41.8%
*Mobile services* ^†^	125	14.7%	79	12.8%	204	26.5%
*Research / surveillance*	33	3.9%	30	4.9%	63	8.2%
*Other*	7	0.8%	11	1.8%	18	2.3%
No answer	94	11.1%	69	11.2%	163	21.2%
**Exposed to tailored HIV interventions (past 12 months)**						
*No*	1,061	74.6%	806	74.5%	1,867	74.6%
*Yes*	272	19.1%	196	18.1%	468	18.7%
*No answer*	89	6.3%	80	7.4%	169	6.7%

*Among those who reported ever been tested: MSM, N = 849; TGW, N = 616.

^†^Managed by MSM and TGW community-based organizations.

TRC: Thai Red Cross.

When compared with face-to-face recruitment, the majority of participants recruited through social media opted for unassisted HIVST (71.8% vs. 2.4% for MSM; 51.0% vs.2.5% for TGW) (Table 5). Social media participants who opted for unassisted HIVST also largely preferred express mail service to obtain their kits (77.6% of MSM, 56.6% of TGW). Conversely, participants who were recruited face to face were more likely to choose assisted HIVST (97.3% vs. 26.1% for MSM, 96.8% vs. 48.6% for TGW).

**Table 5:** Testing modalities by mode of recruitment.

	MSM				TGW				Total			
	Face to Face		Social Media		Face to Face		Social Media		Face to Face		Social Media	
	n	%	n	%	n	%	n	%	n	%	n	%
**Total participants (N)**	1,092	100%	330	100%	874	100%	208	100%	1,966	100%	538	100%
**Selected HIV testing options**												
*Assisted HIVST*	1,062	97.3%	86	26.1%	846	96.8%	101	48.6%	1,908	97.0%	187	34.8%
*Unassisted HIVST*	26	2.4%	237	71.8%	22	2.5%	106	51.0%	48	2.4%	343	63.8%
*Referral to HTS*	4	0.4%	7	2.1%	6	0.7%	1	0.5%	10	0.5%	8	1.5%
**Selected kit delivery options for unassisted HIVST** *												
*POW in the community*	11	42.3%	25	10.5%	16	72.7%	34	32.1%	27	56.3%	59	17.2%
*CBO facility*	9	34.6%	28	11.8%	6	27.3%	12	11.3%	15	31.3%	40	11.7%
*Express mail service*	6	23.1%	184	77.6%	0	0.0%	60	56.6%	6	12.5%	244	71.1%

*Among those who selected unassisted HIVST.

HIVST: HIV self-testing; HTS: HIV testing services; POW: peer outreach worker; CBO: community-based organization.

Taking into account only data from unassisted and assisted HIVST, MSM first-time testers were more likely to select assisted HIVST when compared to repeat testers: 84.5% (415/491) for first-time testers versus 78.8% (667/849) for repeat testers. For TGW, no significant difference was found between first-time and repeat testers: 88.9% (368/414) of first-time testers selected assisted HIVST compared to 87.3% (538/616) of repeat testers.

### Case finding

Case finding varied significantly by HIV testing modality for both MSM and TGW (Table 6). Taking into account only data from unassisted and assisted HIVST and for those who had a reactive or negative HIVST result, the reactivity rate was higher among MSM who opted for unassisted HIVST compared to assisted HIVST: 9.6% (25/261) versus 6.2% (71/1,144), respectively. Inversely, the reactivity rate was higher among TGW who opted for assisted HIVST compared to unassisted HIVST: 7.3% (69/946) and 2.4% (3/124), respectively. Using the same data subset, TGW first-time testers were significantly more likely to have a reactive result compared with repeat testers: 8.1% (33/409) versus 5.0% (31/616), respectively. However, the difference was not substantial for MSM: 7.4% (36/488) of first-time testers had a reactive result versus 6.2% (52/841) of repeat testers. In the referral option under which participants were tested using the national HIV testing algorithm at HIV testing services, one MSM out of 11 (9.1%) and one TGW out of seven (14.3%) tested HIV positive. Participants with reactive or invalid HIVST test results as well as unassisted HIVST participants who reported being unable to interpret the result were referred to HIV testing services for a confirmatory test following the national HIV testing algorithm. Among all participants who needed a confirmatory test (n = 179), 108 (60.3%) were referred to and accessed HIV testing services.

**Table 6:** Outcomes of HIV testing by testing modality.

	MSM						TGW					
	Assisted		Unassisted		Referral		Assisted		Unassisted		Referral	
	HIVST		HIVST				HIVST		HIVST			
	n	%	n	%	n	%	n	%	n	%	n	%
**Total participants (N)**	1,148	100%	263	100%	11	100%	947	100%	128	100%	7	100%
**HIVST and HIV test results**												
*Negative*	1,073	93.5%	236	89.7%	10	90.9%	877	92.6%	121	94.5%	6	85.7%
*Reactive* * */ Positive* ^†^	71	6.2%	25	9.5%	1	9.1%	69	7.3%	3	2.3%	1	14.3%
*Invalid*	4	0.3%	2	0.8%	0	0.0%	1	0.1%	0	0.0%	0	0.0%
*I can’t interpret result*	0	0.0%	0	0.0%	0	0.0%	0	0.0%	4	3.1%	0	0.0%
**Linkage to HTC services for HIV confirmatory test** ^‡^												
*Linked to HTS services*	42	56.0%	17	63.0%	N/A	N/A	48	68.6%	1	14.3%	N/A	N/A
*Lost to follow-up*	33	44.0%	10	37.0%	N/A	N/A	22	31.4%	6	85.7%	N/A	N/A
**HIV confirmatory test results** ^§^												
*Negative*	3	7.1%	1	5.9%	N/A	N/A	1	2.1%	1	100%	N/A	N/A
*Positive*	39	92.9%	16	94.1%	N/A	N/A	47	97.9%	0	0.0%	N/A	N/A
**Linkage to ART services** ^¶^												
*Linked to ART services*	34	87.2%	16	100%	1	100%	39	83.0%	0	0.0%	1	100%
*Lost to follow-up*	5	12.8%	0	0.0%	0	0.0%	8	17.0%	0	0.0%	0	0.0%

*Reactive test result for assisted and unassisted HIVST—not confirmed yet.

^†^Positive HIV testing result (national HIV testing algorithm) for referral to facility only.

^‡^Among those who had assisted or unassisted HIVST with a reactive or invalid HIVST result or who reported "I can’t interpret result".

^§^Among those who accessed HTC services for HIV confirmatory test (national HIV testing algorithm).

^¶^Among those who were confirmed or directly tested (referral option) HIV positive at HTC services.

HIVST: HIV self-testing; HTS: HIV testing services; F: Fischer Test; N/A: Not available; ART: antiretroviral therapy.

### Links to HIV testing and treatment services

Successful links to HIV testing services varied across HIVST modalities and populations (Table 6). For MSM who needed a confirmatory HIV test, linkage to HIV testing services was higher for those who selected unassisted HIVST: 63.0% (17/27) versus 56.0% (42/75) for assisted HIVST. However, for TGW, successful linkage to HIV testing services was significantly higher for those who selected assisted HIVST compared with those who selected unassisted HIVST: 68.6% (48/70) and 14.3% (1/ 7), respectively.

The majority of MSM and TGW who accessed confirmatory HIV testing services preferred receiving these services from CBOs managed by MSM and TGW: of 108 participants successfully linked to HIV testing services, 77 (71.3%) chose a CBO (69.5% [41/59] MSM; 73.5% [36/49] TGW), 19 (17.6%) chose governmental services, 11 (10.2%) a private clinic, and one (0.9%) went to the Thai Red Cross Anonymous Clinic in Bangkok.

All participants who had a reactive HIVST result and were successfully linked to HIV testing services received a confirmed HIV positive test result, but one was confirmed HIV negative (false reactive). Additionally, five participants who had an invalid HIVST result were confirmed HIV negative. The majority of MSM and TGW who were confirmed HIV positive at HIV testing services were linked to treatment services (91 of 104, 87.5%). The link-to-treatment-services rate was somewhat similar for MSM and TGW: of 56 MSM confirmed HIV positive, 51 (91.1%) were linked; of 48 TGW, 40 (83.3%) were linked.

### Reports of adverse events

A total of 19 participants out of 2,451 (0.8%) reported at least one adverse event within 48 hours after an unassisted or assisted HIVST. The most frequent event reported was emotional or cognitive stress: 18 of 2,451 participants (0.7%). No severe adverse events were identified during the interviews conducted by the counselors within 48 hours after HIVST or during routine study monitoring.

Participants who reported any adverse event received follow-up calls from community-based counselors to discuss the situation, and no referrals to mental health services were made as all participants regained their previous mental health status after several conversations with their counselor.

### Intention to use HIVST and preferred kit delivery points

Very few participants—21 of 1,422 MSM (1.5%) and 11 of 1,082 TGW (1.0%)—indicated no interest in HIVST in the future (Table 7). About two-thirds of MSM (945 of 1,422, 66.5%) and TGW (752 of 1,082, 69.5%) reported interest in oral fluid-based HIVST in the future while a small minority (70 of 1,422 MSM [4.9%]; 51 of 1,082 TGW [4.7%]) declared they would prefer blood-based HIVST (finger prick). Some participants were keen to use either oral fluid-based or blood-based HIVST (Table 7).

**Table 7:** Intention to use HIVST and preferred HIVST kit delivery points.

	MSM		TGW		Total	
	*n*	*%*	*n*	*%*	*n*	*%*
**Total participants (N)**	1,422	100%	1,082	100%	2,504	100%
**Preferred HIVST modality for next HIVST**						
*Oral fluid-based*	945	66.5%	752	69.5%	1,697	67.8%
*Finger prick (capillary blood)*	70	4.9%	51	4.7%	121	4.8%
*Any modality*	211	14.8%	122	11.3%	333	13.3%
*Not interested in HIVST*	21	1.5%	11	1.0%	32	1.3%
No answer	175	12.3%	146	13.5%	321	12.8%
**Intention to use unassisted HIVST**						
*No*	479	33.7%	405	37.4%	884	35.3%
*Yes*	801	56.3%	575	53.1%	1,376	55.0%
*No answer*	142	1.0%	102	9.4%	243	9.7%
**Preferred delivery point of HIVST kit in the future** *						
*CBO facility*	303	24.7%	209	22.6%	512	23.8%
*Governmental health facility*	175	14.3%	132	14.3%	307	14.3%
*Pharmacy*	345	28.1%	296	32.0%	641	29.8%
*Private health facility*	81	6.6%	64	6.9%	145	6.7%
*During peer outreach activities*	109	8.9%	103	11.1%	212	9.9%
*By mail service via CBO*	49	4.0%	17	1.8%	66	3.1%
*By mail service via online store*	71	5.8%	21	2.3%	92	4.3%
*Convenience store*	49	4.0%	38	4.1%	87	4.0%
*Other*	4	0.3%	1	0.1%	5	0.2%
*No answer / I don’t know*	40	3.3%	44	4.8%	84	3.9%
**Intention to recommend unassisted HIVST to their peers** ^†^						
*Extremely unlikely*	0	0.0%	3	2.3%	3	0.8%
*Unlikely*	5	1.9%	0	0.0%	5	1.3%
*Neutral*	10	3.8%	6	4.7%	16	4.1%
*Likely*	90	34.2%	37	28.9%	127	32.5%
*Extremely likely*	151	57.4%	77	60.2%	228	58.3%
*No answer*	7	2.7%	5	3.9%	12	3.1%

*Among those interested in oral fluid, finger prick, or any HIVST modality: MSM, N = 1,226; TGW, N = 925.

^**†**^Among those who had unassisted HIVST only: MSM, N = 263; TGW, N = 128.

HIVST: HIV self-testing; CBO: community-based organization.

We also explored participants’ intention to use unassisted HIVST. Out of 1,422 MSM, 801 (56.3%) and out of 1,082 TGW, 575 (53.1%) indicated they would use unassisted HIVST, with participants under the age of 25 significantly more likely than those age 25 or older: 62.9% (771/1,226) versus 58.5% (605/1,034). About half of participants who selected assisted HIVST in this study (1,101 of 2,095, 52.6%) reported their intention to use unassisted HIVST in the future compared with two-thirds of those who opted for unassisted (271 of 391, 69.3%). Among the 391 MSM and TGW participants who selected unassisted HIVST in this study, 228 (58.3%) and 127 (32.5%) reported they were extremely or likely willing, respectively, to recommend this testing modality to their friends (Table 7).

Among participants interested in using either oral fluid-based or blood-based HIVST in the future, about one-fourth of MSM (345 of 1,226, 28.1%) and one-third of TGW (296 of 925, 32.0%) preferred to access the test kit at a pharmacy. Nevertheless, CBOs and governmental health facilities were among the top three choices for delivery points (Table 7). Furthermore, when aggregating all the proposed community-based options for delivery (i.e., community-based facility, peer outreach activities, and mail service via CBOs), the outcome suggests that participants preferred a CBO-led option over all other choices, including pharmacies: 461 of 1,422 MSM (32.4%) and 329 of 1,082 TGW (30.4%) mentioned preferring some community-based delivery model (Table 7).

### Willingness to pay for HIVST kit

**Fig 1:**
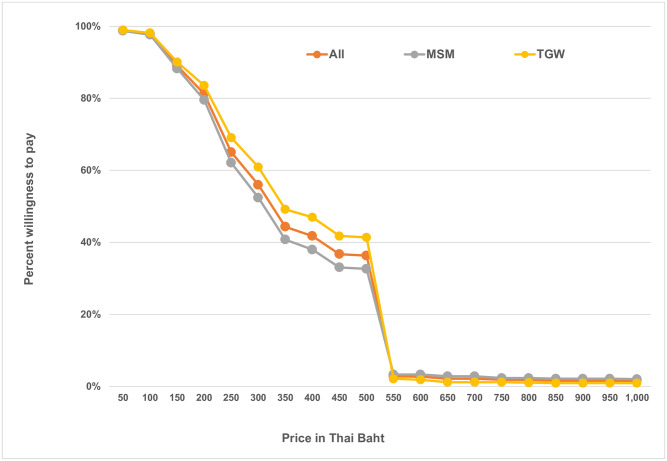
Willingness to pay for HIVST among potential MSM and TGW users.

We assessed the willingness to pay for an HIVST kit among MSM and TGW participants who reported their interest in using oral fluid-based or blood-based HIVST in the future and who also responded to questions regarding willingness to pay in the questionnaire (1,032 MSM and 754 TGW). Almost all participants (98.7% of MSM and 98.9% of TGW) reported they were willing to pay at least 50 Thai baht (about US$1.50 in 2017) for an HIVST kit (Fig 1). The median price they would be willing to pay was 300 Thai baht (about US$9.30), with an interquartile range of 300 Thai baht for both groups. Roughly two-fifths (41.8%) of participants were willing to pay 400 Thai baht (about US$12.40), the projected price for an OraQuick^®^ test kit once marketed in Thailand. Nevertheless, data from this analysis (Fig 1) suggest that the most suitable price for an HIVST kit would be between 250 and 300 Thai baht (US$7.70–$9.30). If the price were too high, around three-fourths of participants (1,332/1,786, 74.6%) said they would rather get tested at a community-based or government facility offering no-cost HIV testing services.

## Discussion

The findings of this study provide evidence of high uptake and level of acceptability of oral fluid-based HIVST among MSM and TGW in Thailand, in line with numerous worldwide studies with key populations [15, 25, 26]. The high level of intention of the participants to recommend unassisted HIVST to their social networks suggests that many participants had positive feelings about their first experience with HIVST. The findings also indicate that HIVST attracted specific segments of MSM and TGW populations that have been generally more difficult to serve with standard HIV testing options. The majority of participants in this study reported no prior exposure to HIV interventions in the last 12 months, and slightly more than one-third reported no previous history of HIV testing—the majority of these selected oral fluid-based HIVST. Furthermore, more than half of participants were under age 25, a group that has been more difficult to reach with facility-based testing services [27, 28]. Finally, data showed that the majority of participants recruited and enrolled through social media opted for unassisted HIVST and—among online MSM specifically—most opted to receive their test kit via express mail instead of in-person contact. This suggests the study captured traditionally hard-to-reach MSM and TGW who prefer not to interact directly with service providers at community-based or health care facilities.

Assisted HIVST was the most favored option selected by both populations compared to unassisted HIVST and standard referral to HIV testing services. About one-third of the participants reported they would not opt for unassisted HIVST in the future. These findings suggest that unassisted and assisted HIVST are complementary and serve the needs of different segments of the MSM and TGW populations in Thailand. We observed that about one-fifth (20%) of the participants reported preferring only capillary blood-based or both capillary blood- and oral fluid-based HIVST modalities. Capillary blood-based rapid diagnostic tests have a higher sensitivity compared to oral fluid-based HIV tests [14] and could be considered as a valuable tool to follow up clients with specific needs under the PrEP program in Thailand.

MSM and TGW in Thailand are willing to pay for HIVST kits, if priced reasonably. A price ranging between US$7.70 and $9.30 would ensure that kits are affordable and perceived to be trusted by the majority of MSM and TGW participants. The finding on willingness to pay is consistent with data from studies conducted in middle-income countries [15], and the finding on price is slightly lower than the charge OraSure is expecting to use to market the kit in Thailand (about US$12.00). If OraSure maintained this price for Thailand, subsidization would be required to make the kits affordable. Since participants identified CBOs and pharmacies as the most preferred delivery points for HIVST kits, it would be critical, for ensuring access to HIVST for different segments of these populations, to explore the possibility of establishing a social marketing strategy, including revolving funds, with selected organizations and pharmacy stores, particularly if the market price for the kit established by the manufacturer were above US$10.00.

Case finding (e.g., participants with reactive HIVST results) varied across populations and HIVST modalities, with a higher reactivity rate among MSM who had unassisted HIVST compared to assisted HIVST. However, this was the opposite for TGW participants where the reactivity rate was higher for those who had assisted HIVST. Despite systematic follow-up contacts with concerned participants (e.g., those who had a reactive or invalid HIVST result or could not interpret their HIVST result), linkage to HIV testing services remained suboptimal (60% overall). Informal discussions with peer outreach workers during monitoring activities and dissemination of preliminary findings in the community indicated that most of those who were lost to follow-up either asked peer outreach workers to halt follow-up calls for privacy reasons, stopped responding to follow-up calls, or had telephone numbers that became unavailable. A consensus emerged from these discussions that these cases were definitively lost to follow-up for the study, but participants probably accessed HIV testing services from a desired location outside the referral network of the study so as not to be in doubt concerning their final HIV status. Further investigations would be needed to understand the factors predicting HIVST test-seeking behaviors to improve linkage rates and increase individual and public health benefits of HIVST. These efforts should explore barriers to access to confirmatory testing in more detail, as well as attitudes toward potential solutions—such as mobile, in-home, or by mail confirmatory testing—to overcome these barriers. Peer outreach workers also noted that among participants who were successfully followed up some were linked to HIV testing services immediately after or within a few days of obtaining their HIVST result, while others required numerous follow-up calls spread over several weeks to convince them to access confirmatory HIV testing. Among those who were confirmed HIV positive, across populations and testing modalities (HIVST and referral options), more than 80% were successfully linked to treatment services. Among those not linked to treatment, informal discussion with outreach workers highlighted challenges similar to those linking reactive clients to confirmatory testing.

Notably, no serious adverse events were reported by participants who conducted either unassisted or assisted HIVST. However, some participants, particularly those who screened reactive, reported minor mental health issues similar to those reported in studies of people diagnosed HIV positive through facility-based testing services [29–33]; thus, these outcomes should not be attributed to self-testing modalities specifically. Systematic follow-up after HIVST helped the research team quickly identify and manage these issues and may have prevented the occurrence of severe adverse events.

This study had some limitations. First, a convenience sampling was used, affecting the representativeness of the sample and limiting the extrapolation of these findings to all MSM and TGW populations in Thailand. Second, while reliable data on positivity rates and linkage to services are critical for measuring individual and public health benefits of HIVST and optimizing implementation of HIV interventions, this study relied on self-reported linkage to service; however, some participants may have falsely reported accessing confirmatory testing or treatment services to avoid follow-up and to protect their privacy. The rates of successful linkage to services may therefore be overestimated [34]. However, it would be challenging for implementers to obtain more reliable data without jeopardizing the privacy and confidentiality of individuals, which participants in this study identified as the key benefits that made HIVST attractive in the first place.

## Conclusions

This study demonstrated a high uptake of oral fluid-based HIVST in conjunction with a notable level of willingness to pay for HIVST kits. Findings highlight that HIVST is safe, as no adverse events were found, and the need to offer both assisted and unassisted self-testing models. MSM and TGW surveyed in this study also highlighted the critical role of CBOs and pharmacies as the relevant delivery points for HIVST. The preliminary findings of this study also provided a strong basis for the Thai Ministry of Public Health (MOPH) to issue the country’s first HIVST policy in April 2019, which they refined based on several rounds of dialogue with key stakeholders, including CBOs that participated in the preparation and implementation of this study. Manufacturers of HIVST kits are currently working with the Thai Food and Drug Administration (FDA) on the registration of HIVST kits, with the first kit expected to be successfully registered in the middle of 2021. HIVST should then be scaled up by investing in support mechanisms for high-risk populations.

## Supporting information

S1 Text Questionnaire in English for quantitative data collection. (PDF)

S2 Text Questionnaire in Thai for quantitative data collection. (PDF)

S1 Dataset De-identified data set used for data analyses reported in this study. (XLSX)

S1 Codebook Codebook of the data set used for data analyses reported in this study. (PDF)
