# Supplementary material for: Uptake of oral fluid-based HIV self-testing among men who have sex with men and transgender women in Thailand
Source: PLoS One. 2021 Aug 16;16(8):e0256094. doi: 10.1371/journal.pone.0256094 (PMC8367007; doi:10.1371/journal.pone.0256094)
Supplement: S1 Text — (PDF) [file pone.0256094.s001.pdf]

QUESTIONNAIRE [QUANTITATIVE]

ALL PARTICIPANTS

This questionnaire will be formatted for the CommCare application and mobile website

<u>INTRODUCTION</u>: Thank you for agreeing to take part in this important study and for completing this questionnaire. Be assured that all answers you provide will be kept in the strictest confidentiality. Your answers in this questionnaire will help us to understand your opinion and/or experience with regard to HIV oral fluid testing, particularly HIV self-testing. The results for this study will help the Thai Ministry of Public Health to develop new policy on HIV self-testing. It should take 40-45 minutes to complete this questionnaire.

If you do not feel like to answer to one question, please tick “no answer” to go to the next question. Please click “NEXT” to begin.

Section 1: Socio-demographic characteristics

1.1 What is your age and year of birth?

1.1.1 Age: __ ___ [99 – No answer]
1.1.2: Year of birth: __ __ __ __ (YYYY- Buddhist calendar) [99- No answer]

1.2 Self-reported gender

(1) Male
(2) Female
(3) Transgender
(4) Undecided
(99) No answer

1.3 In past 12 months, have you had sex (vaginal or anal or oral sex) with any of the following partner?

(1) A partner born with male body parts
(2) A partner born with female body parts
(3) Both types of partner
(99) No answer

1.4 Highest level of education you have completed

(0) No schooling or not completed primary level (P6)
(1) Primary (completed P1 – P6)
(2) Secondary (completed M1 – M3)
(3) High-school (completed M4 – M6)
(4) University or higher (completed at least one university degree)
(99) No answer

1.5 Current Occupation

(0) Unemployed
(1) Student
(2) Civil servant (including military/police)
(3) Self-employee
(4) Employee outside government
(5) Other: specify ________________________
(99) No answer

1.6 Current marital status

(1) Married to a woman
(2) Divorced – separated
(3) Widowed
(4) Single
(99) No answer

1.7 Number of children

[99 – No Answer]

1.8 You are currently living with

(1) Alone
(2) With friends
(3) With family members/relatives
(4) With wife or regular female partner
(5) With regular male partner
(99) No answer

1. 9 Average monthly income from any work done or services provided in past year

###, ### THB [99 – No answer]

1.10 For how long have you been living in this city?

(1) I am a visitor – I don't leave in this city
(2) Less than 1 month
(3) 1 – 12 months
(4) > 12 months
(99) No answer

Section 2: Sexual behaviors in past 3 months

2.1 Did you use condoms during the last vaginal or anal sex with your wife or female (not a transgender) regular partner — a person with whom you have emotional bond?

(0) No
(1) Yes
(2) I don't have a wife or female regular partner
(99) No answer

2.2 Did you use condoms during the last vaginal or anal sex with a female casual or sex worker partner (not a transgender)?

(0) No
(1) Yes
(2) I did not have a female casual or sex worker partner
(99) No answer

2.3 Did you use condoms during the last anal sex with your male (or transgender) regular partner — a person with whom you have emotional bond?

(0) No
(1) Yes
(2) I did not have a male regular partner
(99) No answer

2.4 Did you use condom during the last anal sex with your male (or transgender) casual/sex worker/client partner?

(0) No
(1) Yes
(2) I did not have a male casual partner
(99) No answer

2.5 How often have you used condoms in past 3 months during anal sex with any male (or transgender) partners?

(0) Never
(1) Sometimes
(2) Most of the time
(3) Always
(4) No anal sex in past 3 months
(99) No answer

2.6 What was your role during anal sex (not oral) with any male partners in past 3 months?

(1) Always insertive
(2) Sometimes insertive and sometime receptive
(3) Always receptive
(4) No anal sex in past 3 months
(99) No answer

2.7 The last time you had a non-regular male sexual partner, where did you first meet/contact him? (One answer)

(1) Social media
(2) Referred by a friend
(3) Private party (excluding sex party)
(4) Private sex party
(5) Bar/discotheque
(6) Sauna/massage parlor
(7) In open-air public place (such as street, public park, shopping mall, beach, etc.)
(8) Other: specify ________________
(99) No answer

2.8 In the past 3 months, have you received from another male (or transgender) partner, money or goods in exchange for sexual services?

(0) No
(1) Yes
(99) No answer

2. 9 In the past 3 months, have you given to another male (or transgender) partner, money of goods in exchange for sexual services?

(0) No
(1) Yes
(99) No answer

Section 3: Health seeking behaviors for HIV testing

3.1 When was the last time you took an HIV test and received the result?

(0) Never been tested
(1) Less than 3 months ago
(2) Between 3- 6 months
(3) Between 6 – 12 months
(4) > 12 months
(99) No answer

3.2 In the past 12 months, how often have you been tested for HIV test and received the result?

(0) I haven't been tested for HIV in past 12 months
(1) 1 time
(2) 2 times
(3) 3 times
(4) > 3 times
(99) No answer

3.3 The last time you took an HIV test and received the result, where was it?

(0) Never been tested
(1) At Clinic at the DIC
(2) At private clinic/hospital
(3) At Anonymous clinic of Thai Red Cross
(4) At governmental hospital
(5) During mobile services provides by CBOs and/or GOs
(6) During a research/surveillance
(7) Other: specify ________________________
(99) No answer

3.4 In order to understand the quality of the services you received the last time you had an HIV test, rate your level of agreement of the following items by recording a number from 1 to 10 using the scale given below. If you have never been tested, record the number "0" in all the items below:

	1	***2***	***3***	***4***	***5***	***6***	***7***	***8***	***9***	***10***	***0***	***99***
Strongly Disagree										***Strongly Agree***	***Never been Tested***	***No answer***

<u>Items</u>: I feel that…

3.4.1 The opening time of the clinic or the time the service was provided was convenient: ##
3.4.2 My privacy was respected: ##
3.4.3 The care providers were friendly: ##
3.4.4 I did not wait for a long time before seeing the care provider: ##
3.4.5 The blood collection was not painful: ##
3.4.6 I had the results within one hour after my blood was collected: ##
3.4.7 I received clear and relevant information for HIV and STI prevention: ##
3.4.8 The results will be kept confidential: ##

Section 4: Stigma/Discrimination

4.1 Have you ever been verbally abused (insulted) because of your sexual orientation or behavior?

(0) Never
(1) Yes, in past 12 months
(2) Yes, more than 12 months ago
(99) No answer

4.2 Have you ever been physically attacked because of your sexual orientation or behavior?

(0) Never
(1) Yes, in past 12 months
(2) Yes, more than 12 months ago
(99) No answer

4.3 Have you ever been refused health services because of your sexual orientation or behavior?

(0) Never
(1) Yes, in past 12 months
(2) Yes, more than 12 months ago
(99) No answer

4.4 In the past year have you avoided accessing health services because you were afraid to be discriminated/judged on your sexual orientation or behavior?

(0) Never
(1) Yes, in past 12 months
(2) Yes, more than 12 months ago
(99) No answer

4.5 In general, do you feel embarrassed to disclose and discuss your sexual orientation or behavior to a care provider at a health facility?

(1) Not at all embarrassed
(2) Slightly embarrassed
(3) Moderately embarrassed
(4) Very embarrassed
(5) Extremely embarrassed
(99) No answer

4.6 To understand the ways you may feel if you were diagnosed with HIV, rate your level of agreement of the following items by recording a number from 1 to 10 using the scale given below.

1	***2***	***3***	***4***	***5***	***6***	***7***	***8***	***9***	***10***	***99***
Strongly Disagree									***Strongly Agree***	***No answer***

<u>Items</u>: I think that if I were diagnosed HIV positive…
4.6.1 It would be difficult to tell people about my HIV infection: ##
4.6.2 It would make feel dirty: ##
4.6.3 I would feel guilty: ##
4.6.4 I would feel ashamed: ##
4.6.5 I would feel worthless: ##

4.6.6 I would hide my HV status from other: ##

Section 5: Knowledge/Awareness/Experience with self-testing

5.1 Could you be infected with HIV if you have unprotected anal sex (insertive or receptive) with another male partners?

(0) No
(1) Yes
(97) I don't know
(99) No answer

5.2 Are there drugs available in Thailand for helping people living with HIV to stay healthy and live/work normally?

(0) No
(1) Yes
(97) I don't know
(99) No answer

5.3 Do you think that these drugs are accessible for all Thai citizens through the Thai social welfare scheme?

(0) No
(1) Yes
(97) I don't know
(99) No answer

5.4 Have you ever heard/read about HIV self-testing using oral fluid before being contacted for this study?

(0) No
(1) Yes
(99) No answer

5.5 Have you ever heard/read about HIV self-testing using blood from a finger prick?

(0) No
(1) Yes
(99) No answer

5.6 Have you ever done an HIV-self-testing using oral fluid or using blood from a finger prick before?

(0) Never
(1) Yes using oral fluid
(2) Yes using blood from finger prick
(99) No answer

Section 6: Perceived concerns and benefits

6.1 How common is HIV infection among MSM/transgender women in Thailand?

(1) Very uncommon
(2) Somewhat uncommon
(3) Neither common or uncommon
(4) Somewhat common
(5) Very common
(97) I don't know
(99) No answer

6.2 Do you personally know anyone who is infected with HIV?

(0) No
(1) Yes
(99) No answer

6.3 How likely are you to contract HIV?

(1) Extremely unlikely
(2) Unlikely
(3) Neutral
(4) Likely
(5) Extremely likely
(97) I don't know
(99) No answer

6.4 How serious would it be if you were infected with HIV?

(1) Not at all serious
(2) Slightly serious
(3) Neither
(4) Somewhat serious
(5) Extremely serious
(97) I don't know
(99) No answer

6.5 How easy would it be to get an HIV test if you wanted one?

(1) Very difficult
(2) Somewhat difficult
(3) Neither difficult or easy
(4) Fairly easy
(5) Very easy
(97) I don't know
(99) No answer

6.6 Do you think that there are benefits for you to know your HIV status?

(1) Strongly disagree
(2) Disagree
(3) Neither disagree or agree
(4) Agree
(5) Strongly agree
(97) I don't know
(99) No answer

6.7 How easy is it to get treatment in Thailand if you are infected with HIV?

(1) Very difficult
(2) Somewhat difficult
(3) Neither difficult or easy
(4) Fairly easy
(5) Very easy
(97) I don't know
(99) No answer

Section 7: Intention/self-efficacy/willingness to pay

7.1 If you had access to an HIV home test kit, would you increase the frequency of HIV testing?

(1) Extremely unlikely
(2) Unlikely
(3) Neutral
(4) Likely
(5) Extremely likely
(97) I don't know
(99) No answer

7.2 If you wanted to use an HIV oral fluid or blood-based self-testing kit (i.e., collecting oral fluid from your gums or blood from your finger and interpreting the results yourself) which will include instructions in Thai language and pictures to help you to perform the test and the contact of a care provider to discuss the results of the test or if you have any questions/concerns, how certain are you that you can execute the tasks below by yourself? Rate your degree of confidence by recording a number from 1 to 10 using the scale given below:

	1	2	3	4	5	6	7	8	9	10	99
Cannot do at all										***Highly certain can do***	***No answer***

<u>**Tasks**</u>

7.2.1 Requesting the HIV self-testing kit at a delivery point: ##
7.2.2 Following the instructions to perform the test: ##
7.2.3 Collecting oral fluid (fluid on your gum, not saliva) using a swab: ##
7.2.4 Collecting drops of blood from your finger with a small needle: ##
7.2.5 Interpreting the results: ##
7.2.6 Contacting a care provider to discuss the results: ##
7.2.7 Going to a clinic to get an HIV test for confirmation if you test positive with the self-test kit: ##
7.2.8 Going to a HIV care and treatment clinic if your HIV test result was confirmed positive: ##

7.3 Would you be willing to pay 400 THB for buying an HIV self-testing kit?

(0) No
(1) Yes
(97) I don't know
(99) No answer

7.4 Would you be willing to pay for the HIV self-testing kit if the price increases to 500 THB?

(0) No
(1) Yes
(97) I don't know
(99) No answer

7.5 For buying an HIV self-testing kit, would you be willing to pay for:

(1) A lower increase
(2) A higher increase
(97) I don't know
(99) No answer

7.6 What is highest price you would pay for buying a HIV self-test kit?

### THB [99 – No answer]

7.7 What would you do if the price for an HIV self-testing kit were too high?

(1) I will not have an HIV test
(2) Go to get free HIV testing at HIV testing and counseling services at government health facility or community based organizations
(3) Go to private services and paying for HIV testing by myself
(97) I don't know
(99) No answer

7.8 If you wanted to use a self-testing kit, what would be for you <u>the best point</u> of delivery to get the kit? (One answer only)

(1) Community-based organization
(2) At governmental health facility
(3) At drug store/pharmacy
(4) Private clinics
(5) Community-based supporters during outreach
(6) Through surface express mail (EMS) after ordering from a CBO
(7) Through surface express mail (EMS) after ordering from Internet
(8) A 7/11-type shop
(9) Sauna, bar or massage parlors
(10) Other: specify ___________________________
(97) I don't know
(99) No answer

7.9 What would be the preference if you decided to do an HIV self-testing?
(One answer only)

(1) Using an oral fluid self-testing kit (swabbing the gums to collect oral fluid)
(2) Using a blood-based self-testing kit (taking drops of blood from your finger with a lancet)
(3) Either oral fluid or blood-based
(4) I am not interested in doing an HIV-self-test
(97) I don't know
(99) No answer

Section 8: Substance use

8.1 Have you ever injected recreational drugs (for pleasure/fun, not medicine prescribed by physician)?

(0) Never
(1) Yes, in the past 3 months
(2) Yes, between 3-12 months ago
(3) Yes, more than one year ago
(99) No answer

8.2 Have you ever swallowed/smoked/snorted recreational drugs (for pleasure/fun, not medicine prescribed by physician)?

(0) Never
(1) Yes, in the past 3 months
(2) Yes, between 3-12 months
(3) Yes, more than one year ago
(99) No answer

8.3 If any drugs recreational were used in past three months, indicate which one:

Multiple answers: for each item, please code:

- 0 if not used in past 3 months
- 1 if used in past 3 months
- *99 - No answer*

8.3.1: Marijuana #
8.3.2: Methamphetamine #
8.3.3: Ecstasy #
8.3.4: Cocaine #
8.3.5: Heroine #
8.3.6 Ice #
8.3.7: Barbiturates/benzodiazepines #
8.3.8: Solvent – Glue #
8.3.9: Ketamine #
8.3.10: Poppers #
8.3.11: Other: specify ____________________

8.4 In past 3 months, have you used any drugs for sexual enhancement such as Viagra or Cialis before having sex?

(0) No
(1) Yes
(99) No answer

Section 9: Exposure to interventions

9.1 In past 12 months, how often have you participated in specific activities (face-to-face or small group discussion) to discuss HIV prevention, care and treatment for MSM and/or transgender people?

(0) Never
(1) 1 time
(2) 2 times
(3) 3 times
(4) > 3 times
(97) I don't know
(99) No answer

9.2 During these prevention activities in the past 12 months, have you been referred for an HIV test?

(0) No
(1) Yes
(99) No answer

9.3 Did you as a result of that referral got tested for HIV and learnt your test result?

(0) No
(1) Yes
(2) I was not referred during these activities
(99) No answer

Section 10: Medication history

10. 1 Are you currently taking a long-term medical treatment for any chronic disease?

(0) No
(1) Yes
(99) No answer

10.2 In past 3 months, have you taken/injected any hormones for physical and/or emotional changes?

(0) No
(1) Yes
(99) No answer

10. 3 Have you ever taken Pre-Exposure Prophylaxis (or PrEP) medicines to lower your risk of getting infected with the HIV?

(0) Never
(1) Yes, in the past 2 months
(2) Yes, between 2 – 4 months
(3) Yes, more than 4 months ago
(97) I don’t know or have never heard about PrEP
(99) No answer

10. 4 Have you ever taken Post-Exposure Prophylaxis (or PEP) medicines to reduce the risk of getting infected with HIV after potential exposure such as unprotected anal or vaginal sex

(0) Never
(1) Yes, in the past 2 months
(2) Yes, between 2 – 4 months
(3) Yes, more than 4 months ago
(97) I don’t know or have never heard about PEP
(99) No answer

THE FOLLOWING SECTIONS (SEECTION 11 & 12) ARE ONLY FOR THE PARTCIPANTS WHO SELECT HIV SELF-TESTING AND ANSWER TO THE ONLINE QUESTIONNAIRE

Section 11: Results of the screening test

11.1: The result of the screening test using OraQuick is:

(0) Non-reactive
[If ticked ➔ Message: Seek regular testing. If you may have been exposed to HIV test again in 3 months or in 6 months if not exposed to HIV]

(1) Reactive
[If ticked ➔ Message: You absolutely need to seek additional testing to confirm this result. Contact HIV Testing and Counseling services listed on your study card or on the study's website]

(2) Invalid
[If ticked ➔ Message: You absolutely need to seek additional testing to confirm this result. Contact HIV Testing and Counseling services listed on your study card or on the study's website]

(3) I can't interpret it
[If ticked ➔ Message: You absolutely need to seek additional testing to confirm this result. Contact HIV Testing and Counseling services listed on your study card or on the study's website]

(99) No answer

Section 12: Experience with HIV self-testing using OraQuick

12. 1: Were the written instructions for performing HIV self-testing difficult to understand?

(1) Very difficult to understand
(2) Somewhat difficult to understand
(3) Neither difficult or easy to understand
(4) Fairly easy to understand
(5) Very easy to understand
(97) I did not read the written instructions
(99) No answer

12. 2: Were the instructions explained in the video for performing HIV self-testing difficult to understand?

(1) Very difficult to understand
(2) Somewhat difficult to understand
(3) Neither difficult or easy to understand
(4) Fairly easy to understand
(5) Very easy to understand
(97) I did not watch the video
(99) No answer

12. 3: Was the result of the HIV self-testing difficult to interpret?

(1) Very difficult to interpret
(2) Somewhat difficult to interpret
(3) Neither difficult or easy to interpret
(4) Fairly easy to interpret
(5) Very easy to interpret
(99) No answer

12. 2: Was the HIV self-testing difficult to perform?

(1) Very difficult to perform
(2) Somewhat difficult to perform
(3) Neither difficult or easy to perform
(4) Fairly easy to perform
(5) Very easy to perform
(99) No answer

12. 5: How likely would you be to recommend HIV self-testing using oral fluid to a friend?

(1) Extremely unlikely
(2) Unlikely
(3) Neutral
(4) Likely
(5) Extremely likely
(97) I don't know
(99) No answer
