# Supplementary material for: Uptake of oral fluid-based HIV self-testing among men who have sex with men and transgender women in Thailand
Source: PLoS One. 2021 Aug 16;16(8):e0256094. doi: 10.1371/journal.pone.0256094 (PMC8367007; doi:10.1371/journal.pone.0256094)
Supplement: S2 Text — (PDF) [file pone.0256094.s002.pdf]

แบบสอบถาม (เชิงปริมาณ)

สำหรับอาสาสมัครทุกท่าน

แบบสอบถามชุดนี้จะติดตั้งในระบบคอมแคร์ (CommCare)และเว็ปไซต์บนโทรศัพท์มือถือ

บทนำ: -ขอขอบพระคุณอย่างยิ่งที่ท่านตกลงเข้าร่วมโครงการวิจัยที่สำคัญนี้ และสละเวลาตอบแบบสอบถามชุดนี้ ขอให้ท่านมั่นใจว่าคำตอบของท่านจะถูกเก็บเป็นความลับอย่างเคร่งครัดที่สุด

คำตอบของท่านจะช่วยให้เราเข้าใจความคิดเห็นของท่าน และ/หรือประสบการณ์ของท่านในการตรวจการติดเชื้อเอชไอวีโดยวิธีตรวจจากของเหลวในช่องปาก โดยเฉพาะอย่างยิ่งการตรวจการติดเชื้อเอชไอวีด้วยตนเอง ผลจากการศึกษานี้จะช่วยกระทรวงสาธารณสุขในการพัฒนานโยบายใหม่สำหรับการตรวจการติดเชื้อเอชไอวีด้วยตนเอง การตอบแบบสอบถามชุดนี้ใช้เวลาประมาณ 40-45 นาที

ถ้าท่านไม่ต้องการตอบคำถามข้อใด กรุณาเลือก ‘ไม่ขอตอบ ‘ เพื่อข้ามไปยังคำถามถัดไป

โปรดเลือกปุ่ม ‘ต่อไป’เพื่อเริ่มตอบคำถาม

ส่วนที่ 1: สถานภาพทางสังคม (Socio-demographic)

1.1 กรุณาระบุอายุและปีเกิดของท่าน

1.1.1 อายุ _ _

1.1.2 ปีเกิด_ _ _ _ (พ.ศ.)

1.2 กรุณาระบุเพศของท่าน

(1) ชาย

(2) หญิง

(3) สาวประเภทสอง

(4) ไม่ตัดสินใจ

(99) ไม่ขอตอบ

1.3 ในช่วง 12 เดือนที่ผ่านมา ท่านเคยมีเพศสัมพันธ์ (ทางช่องคลอด หรือทางทวารหนัก หรือทางปาก) กับคู่นอนประเภทใดต่อไปนี้

(1) คู่นอนที่มีเพศกำเนิดเป็นชาย

(2) คู่นอนที่มีเพศกำเนิดปีนหญิง

(3) ทั้งสองแบบ

(99) ไม่ขอตอบ

1.4 ท่านจบการศึกษาสูงสุดระดับใด

(0) ไม่ได้เรียนหนังสือ หรือต่ำกว่าประถมศึกษา (ต่ำกว่าป.6)

(1) ประถมศึกษา (ป.6)

(2) มัธยมศึกษาตอนต้น (ม.3)

(3) มัธยมศึกษาตอนปลาย (ม.6)

(4) มหาวิทยาลัยหรือสูงกว่า (จบอย่างน้อยหนึ่งปริญญา)

(99) ไม่ขอตอบ

1.5 อาชีพหลักในปัจจุบัน

(0) ไม่ได้ทำงาน

(1) นักศึกษา/นักเรียน

(2) ข้าราชการ (รวมทั้งทหารและตำรวจ)

(3) ธุรกิจส่วนตัว

(4) รับจ้าง/พนักงานบริษัทเอกชน

(5) อื่นๆ: โปรดระบุ……………..

(99) ไม่ขอตอบ

1.6 สถานะการสมรสในปัจจุบัน

(1) สมรสกับผู้หญิง

(2) หย่า-แยกกันอยู่

(3) หม้าย

(4) โสด

(99) ไม่ขอตอบ

1.7 จำนวนบุตร............คน

1.8 ปัจจุบันท่านอาศัยอยู่กับ

(1) อยู่คนเดียว

(2) เพื่อน

(3) ครอบครัว / ญาติ

(4) ภรรยา/คู่ที่เป็นหญิง

(5) คู่ที่เป็นชาย

(99) ไม่ขอตอบ

1.9 รายได้เฉลี่ยต่อเดือนจากการทำงานหรือการให้บริการในปีที่ผ่านมา..........................บาท/เดือน

1.10 ท่านอาศัยอยู่ที่จังหวัดนี้มานานเท่าไหร่

(1) มาท่องเที่ยว – ไม่ได้อาศัยอยู่ที่จังหวัดนี้

(2) น้อยกว่า 1 เดือน

(3) ระหว่าง 1-12 เดือน

(4) มากกว่า 12 เดือน

(99) ไม่ขอตอบ

ส่วนที่ 2: พฤติกรรมทางเพศในช่วง 3 เดือนที่ผ่านมา

2.1 ท่านใช้ถุงยางอนามัยหรือไม่ในขณะมีเพศสัมพันธ์ครั้งล่าสุดทางช่องคลอดหรือทวารหนักกับภรรยาหรือคู่นอนประจำ(ที่ไม่ใช่สาวประเภทสอง) ซึ่งเป็นบุคคลที่ท่านมีความผูกพันทางจิตใจและอารมณ์

(0) ไม่ใช้

(1) ใช้

(2) ไม่มีภรรยาหรือคู่นอนประจำเพศหญิง

(99) ไม่ขอตอบ

2.2 ท่านใช้ถุงยางอนามัยหรือไม่ในขณะมีเพศสัมพันธ์ครั้งล่าสุดทางช่องคลอดหรือทางทวารหนักกับคู่นอนชั่วคราวเพศหญิงหรือหญิงขายบริการ (ที่ไม่ใช่สาวประเภทสอง)

(0) ไม่ใช้

(1) ใช้

(2) ไม่มีคู่นอนชั่วคราวที่เป็นหญิงแท้หรือหญิงขายบริการ

(99) ไม่ขอตอบ

2.3 ท่านใช้ถุงยางอนามัยหรือไม่ในขณะมีเพศสัมพันธ์ครั้งล่าสุดทางทวารหนักกับคู่นอนประจำเพศชาย (หรือสาวประเภทสอง) ซึ่งเป็นบุคคลที่ท่านมีความผูกพันทางจิตใจและอารมณ์

(0) ไม่ใช้

(1) ใช้

(2) ไม่มีคู่นอนประจำเพศชาย

(99) ไม่ขอตอบ

2.4 ท่านใช้ถุงยางอนามัยหรือไม่ในขณะมีเพศสัมพันธ์ครั้งล่าสุดทางทวารหนักกับคู่นอนชั่วคราวเพศชาย (หรือสาวประเภทสอง)/ชายขายบริการทางเพศ/ลูกค้า

(0) ไม่ใช้

(1) ใช้

(2) ไม่มีคู่นอนชั่วคราวเพศชาย

(99) ไม่ขอตอบ

2.5 ท่านใช้ถุงยางอนามัยบ่อยแค่ไหนในช่วง 3 เดือนที่ผ่านมาในขณะมีเพศสัมพันธ์ทางทวารหนักกับคู่นอนเพศชายทั่วไป (หรือสาวประเภทสอง)

(0) ไม่เคยใช้

(1) ใช้บางครั้ง

(2) ใช้บ่อยครั้ง

(3) ใช้ทุกครั้ง

(4) ไม่มีเพศสัมพันธ์ทางทวารหนักในช่วง 3 เดือนที่ผ่านมา

(99) ไม่ขอตอบ

2.6 บทบาทของท่านในขณะมีเพศสัมพันธ์ทางทวารหนัก (ไม่ใช่ทางปาก) กับคู่นอนเพศชาย ในช่วง 3 เดือนที่ผ่านมา

(1) เป็นฝ่ายสอดใส่ทุกครั้ง

(2) บางครั้งเป็นฝ่ายสอดใส่ บางครั้งเป็นฝ่ายถูกสอดใส่

(3) เป็นฝ่ายถูกสอดใส่ทุกครั้ง

(4) ไม่มีเพศสัมพันธ์ทางทวารหนักในช่วง 3 เดือนที่ผ่านมา

2.7 ครั้งล่าสุดที่ท่านมีเพศสัมพันธ์กับคู่นอนชั่วคราวเพศชาย ท่านได้พบและติดต่อกับเขาครั้งแรกที่ไหน

(1) สื่อโซเชียล เช่น ลายน์ (LINE) หรือ วอทส์แอ็ป (WhatsApp)

(2) เพื่อนแนะนำ

(3) งานปาร์ตี้ส่วนตัว (ยกเว้นปาร์ตี้เซ็กส์)

(4) งานปาร์ตี้เซ็กส์

(5) บาร์/ ดิสโก้เทค

(6) ซาวน่า/ร้านนวด

(7) ที่สาธารณะ (เช่น ถนน, สวนสาธารณะ, ห้างสรรพสินค้า, ชายหาด ฯลฯ)

(8) อื่นๆ โปรดระบุ…………..

(99) ไม่ขอตอบ

2.8 ในช่วง 3 เดือนที่ผ่านมา ท่าน<u>ได้รับ</u>เงินหรือของขวัญเป็นสิ่งตอบแทนในการมีเพศสัมพันธ์จากคู่นอนเพศชาย (หรือสาวประเภทสอง) หรือไม่

(0) ไม่

(1) ใช่

(99) ไม่ขอตอบ

2.9 ในช่วง 3 เดือนที่ผ่านมา ท่าน<u>ได้ให้</u>เงินหรือของขวัญเป็นสิ่งตอบแทนในการมีเพศสัมพันธ์กับคู่นอนเพศชาย (หรือสาวประเภทสอง) หรือไม่

(0) ไม่

(1) ใช่

(99) ไม่ขอตอบ

ส่วนที่ 3: พฤติกรรมด้านสุขภาพในการตรวจการติดเชื้อเอชไอวี

3.1 ท่านได้ตรวจการติดเชื้อเอชไอวีและได้รับทราบผลครั้งล่าสุดเมื่อใด

(0) ไม่เคยตรวจ

(1) น้อยกว่า 3 เดือน

(2) ระหว่าง 3-6 เดือน

(3) ระหว่าง 6-12 เดือน

(4) มากกว่า 12 เดือน

(99) ไม่ขอตอบ

3.2 ในช่วง 12 เดือนที่ผ่านมา ท่านได้ตรวจการติดเชื้อเอชไอวีและได้ทราบผลด้วยบ่อยแค่ไหน

(0) ไม่ได้ตรวจในช่วง 12 เดือนที่ผ่านมา

(1) 1 ครั้ง

(2) 2 ครั้ง

(3) 3 ครั้ง

(4) มากกว่า 3 ครั้ง

(99) ไม่ขอตอบ

3.3 ครั้งล่าสุดที่ท่านได้ตรวจการติดเชื้อเอชไอวีและได้ทราบผลคือที่ไหน

(0) ไม่เคยตรวจ

(1) คลีนิกในศูนย์ดร็อปอิน

(2) คลีนิกหรือโรงพยาบาลเอกชน

(3) คลีนิกนิรนาม สภากาชาด

(4) โรงพยาบาลของรัฐ

(5) หน่วยบริการเคลื่อนที่ของศูนย์บริการสุขภาพชุมชน และ/หรือ ศูนย์บริการสุขภาพของรัฐ

(6) ในขณะร่วมโครงการวิจัย /การเฝ้าระวังติดตาม

(7) อื่นๆ โปรดระบุ...............

(99) ไม่ขอตอบ

3.4 เพื่อที่จะเข้าใจในคุณภาพของการบริการที่ท่านได้รับการตรวจการติดเชื้อเอชไอวีครั้งล่าสุด กรุณาให้คะแนนระดับความเห็นด้วยของท่านในหัวข้อต่อไปนี้ โดยให้คะแนน 1 ถึง 10 ตามเกณฑ์วัดที่ระบุด้านล่าง ถ้าท่านไม่เคยตรวจ กรุณาใส่ “0” สำหรับทุกข้อ

1 2 3 4 5 6 7 8 9 10 0

ไม่เห้นด้วยอย่างยิ่ง เห็นด้วยอย่างยิ่ง ไม่เคยตรวจ

ฉันรู้สึกว่า...

3.4.1 เวลาเปิดทำการของคลีนิก หรือ เวลาที่ให้บริการสะดวก: _

3.4.2 ฉันได้รับความเคารพในความเป็นส่วนตัว: _

3.4.3 เจ้าหน้าที่มีความเป็นกันเอง: _

3.4.4 ฉันไม่ต้องรอนานที่จะพบเจ้าหน้าที่: _

3.4.5 การเจาะเลือดไม่เจ็บ: _

3.4.6 ฉันได้รับทราบผลภายใน 1 ชั่วโมงหลังจากถูกเจาะเลือด: _

3.4.7 ฉันได้รับข้อมูลที่ชัดเจนในการป้องกันการติดเชื้อเอชไอวีและการการป้องกันการติดโรคทางเพศสัมพันธ์: _

3.4.8 ผลการตรวจถูกเก็บเป็นความลับ: _

ส่วนที่ 4: การตีตรา/การเลือกปฏิบัติ

4.1 ท่านเคยถูกละเมิดทางวาจา (ดูหมิ่น) เนื่องจากวิถีทางเพศหรือพฤติกรรมทางเพศของท่านหรือไม่

(0) ไม่เคย

(1) เคย ในช่วง 12 เดือนที่ผ่านมา

(2) เคย ในช่วงมากกว่า 12 เดือนที่ผ่านมา

(99) ไม่ขอตอบ

4.2 ท่านเคยถูกทำร้ายร่างกายเนื่องจากวิถีทางเพศหรือพฤติกรรมทางเพศของท่านหรือไม่

(0) ไม่เคย

(1) เคย ในช่วง 12 เดือนที่ผ่านมา

(2) เคย ในช่วงมากกว่า 12 เดือนที่ผ่านมา

(99) ไม่ขอตอบ

4.3 ท่านเคยถูกปฏิเสธการให้บริการด้านสุขภาพเนื่องจากวิถีทางเพศหรือพฤติกรรมทางเพศของท่านหรือไม่

(0) ไม่เคย

(1) เคย ในช่วง 12 เดือนที่ผ่านมา

(2) เคย ในช่วงมากกว่า 12 เดือนที่ผ่านมา

(99) ไม่ขอตอบ

4.4.ในปีที่ผ่านมา ท่านหลีกเลี่ยงที่จะใช้บริการสุขภาพเพราะเกรงว่าท่านจะถูกปฏิเสธการให้บริการด้านสุขภาพเนื่องจากวิถีทางเพศหรือพฤติกรรมทางเพศของท่านหรือไม่

(0) ไม่เคย

(1) เคย ในช่วง 12 เดือนที่ผ่านมา

(2) เคย ในช่วงมากกว่า 12 เดือนที่ผ่านมา

(99) ไม่ขอตอบ

4.5 โดยทั่วไป ท่านรู้สึกอายที่จะเปิดเผยหรือพูดถึงเรื่องวิถีทางเพศหรือพฤติกรรมทางเพศของท่านกับเจ้าหน้าที่ศูนย์บริการสุขภาพ ใช่หรือไม่

(1) ไม่รู้สึกอายเลย

(2) รู้สึกอายเล็กน้อย

(3) รู้สึกอายพอประมาณ

(4) รู้สึกอายมาก

(5) รู้สึกอายอย่างยิ่ง

(99) ไม่ขอตอบ

4.6 เพื่อเข้าใจความรู้สึกของท่านที่อาจจะเกิดขึ้นถ้าท่านถูกวินิจฉัยว่าติดเชื้อเอชไอวี กรุณาให้คะแนนระดับความเห็นด้วยของท่านในหัวข้อต่อไปนี้ โดยให้คะแนนจาก 1 ถึง 10 ตามเกณฑ์วัดที่ระบุด้านล่าง

	1	2	3	4	5	6	7	8	9	10	
ไม่เห็นด้วยอย่างยิ่ง											เห็นด้วยอย่างยิ่ง

ฉันคิดว่าถ้าฉันถูกวินิจฉัยว่าติดเชื้อเอชไอวี ....

4.6.1 จะเป็นการยากในการที่จะบอกคนอื่นว่าฉันติดเชื้อเอชไอวี: _

4.6.2 มันทำให้รู้สึกสกปรก: _

4.6.3 ฉันจะรู้สึกผิด: _

4.6.4 ฉันจะรู้สึกละอายใจ: _

4.6.5 ฉันจะรู้สึกไร้ค่า: _

4.6.6 ฉันจะปกปิดไม่ให้คนอื่นทราบสถานะการติดเชื้อเอชไอวี ของฉัน: _

ส่วนที่ 5: ความรู้ /การตระหนัก /ประสบการณ์ในการตรวจเชื้อเอชไอวีด้วยตนเอง

5.1 ท่านสามารถติดเชื้อเอชไอวี ถ้าท่านไม่มีการป้องกันในขณะมีเพศสัมพันธ์ทางทวารหนัก (ไม่ว่ากรณีเป็นฝ่านสอดใส่หรือถูกสอดใส่) กับคู่นอนชาย ใช่หรือไม่

(0) ไม่ใช่

(1) ใช่

(97) ฉันไม่ทราบ

(99) ไม่ขอตอบ

5.2 ในประเทศไทยมียาที่จะช่วยผู้ติดเชื้อเอชไอวีให้สามารถมีสุขภาพแข็งแรงและดำเนินชีวิต/ทำงานได้ตามปกติ ใช่หรือไม่

(0) ไม่ใช่

(1) ใช่

(97) ฉันไม่ทราบ

(99) ไม่ขอตอบ

5.3 ท่านคิดว่าประชาชนไทยสามารถเข้าถึงยาเหล่านี้ทางสวัสดิการสังคม ใช่หรือไม่

(0) ไม่ใช่

(1) ใช่

(97) ฉันไม่ทราบ

(99) ไม่ขอตอบ

5.4 ท่านเคยได้ยินหรืออ่านพบเกี่ยวกับการตรวจการติดเชื้อเอชไอวีโดยวิธีตรวจจากของเหลวในช่องปากก่อนที่จะได้รับการติดติอให้ร่วมโครงการนี้หรือไม่

(0) ไม่เคย

(1) เคย

(99) ไม่ขอตอบ

5.5 ท่านเคยได้ยินหรืออ่านพบเกี่ยวกับการตรวจการติดเชื้อเอชไอวีโดยวิธีเจาะเลือดที่ปลายนิ้วหรือไม่

(0) ไม่เคย

(1) เคย

(99) ไม่ขอตอบ

5.6 ท่านเคยตรวจการติดเชื้อเอชไอวีด้วยตนเองโดยวิธีตรวจจากของเหลวในช่องปากหรือวิธีเจาะเลือดที่ปลายนิ้ว มาก่อนหรือไม่

(0) ไม่เคย

(1) เคย โดยวิธีตรวจจากของเหลวในช่องปาก

(2) เคย โดยวิธีเจาะเลือดที่ปลายนิ้ว

(99) ไม่ขอตอบ

ส่วนที่ 6: การรับรู้เกี่ยวกับข้อกังวลและประโยชน์

6.1 การติดเชื้อเอชไอวีในกลุ่มชายที่มีเพศสัมพันธ์กับชาย หรือ สาวประเภทสองเป็นเรื่องปกติเพียงใดในประเทศไทย

(1) ไม่ใช่เรื่องปกติเลย (ไม่พบทั่วไป)

(2) ไม่ปกติพอสมควร

(3) ไม่ใช่ทั้งปกติ หรือ ไม่ปกติ (กลางๆ)

(4) เป็นเรื่องปกติพอสมควร

(5) เป็นเรื่องปกติมาก (พบทั่วไป)

(97) ฉันไม่ทราบ

(99) ไม่ขอตอบ

6.2 โดยส่วนตัวท่านรู้จักใครที่ติดเชื้อเอชไอวีหรือไม่

(0) ไม่รู้จัก

(1) รู้จัก

(99) ไม่ขอตอบ

6.3 ท่านมีโอกาสติดเชื้อเอชไอวีมากเพียงใด

(1) มีโอกาสน้อยมาก

(2) มีโอกาสน้อย

(3) มีโอกาสกลางๆ

(4) มีโอกาสพอสมควร

(5) มีโอกาสสูงมาก

(97) ฉันไม่ทราบ

(99) ไม่ขอตอบ

6.4 จะมีความร้ายแรงเพียงใดถ้าท่านติดเชื้อเอชไอวี

(1) ไม่ร้ายแรงเลย

(2) ร้ายแรงเล็กน้อย

(3) ร้ายแรงปานกลาง

(4) ร้ายแรงมากพอสมควร

(5) ร้ายแรงมาก

(97) ฉันไม่ทราบ

(99) ไม่ขอตอบ

6.5 จะมีความยากง่ายเพียงใดในการได้รับการตรวจการติดเชื้อเอชไอวี ถ้าท่านต้องการตรวจ

(1) ยากมาก

(2) ยากพอสมควร

(3) ไม่ยากหรือไม่ง่าย

(4) ค่อนข้างง่าย

(5) ง่ายมาก

(97) ฉันไม่ทราบ

(99) ไม่ขอตอบ

6.6 ท่านเห็นด้วยหรือไม่ว่ามีประโยชน์ต่อตัวท่านถ้าท่านทราบสถานะการติดเชื้อเอชไอวี

(1) ไม่เห็นด้วยอย่างยิ่ง

(2) ไม่เห็นด้วย

(3) ไม่ทั้งเห็นด้วยหรือไม่เห็นด้วย

(4) เห็นด้วย

(5) เห็นด้วยอย่างยิ่ง

(97) ฉันไม่ทราบ

(99) ไม่ขอตอบ

6.7 ถ้าท่านติดเชื้อเอชไอวี จะได้รับการรักษาง่ายเพียงใดในประเทศไทย

(1) ยากมาก

(2) ยากพอสมควร

(3) ไม่ยากหรือไม่ง่าย

(4) ค่อนข้างง่าย

(5) ง่ายมาก

(97) ฉันไม่ทราบ

(99) ไม่ขอตอบ

ส่วนที่ 7: ความตั้งใจหรือความสามารถส่วนตัวหรือความเต็มใจที่จะจ่าย

7.1 ถ้าท่านสามารถตรวจการติดเชื้อเอชไอวีที่บ้านได้ ท่านจะเพิ่มความถี่ในการตรวจหรือไม่

(1) ไม่เพิ่มขึ้นแน่นอน

(2) ไม่น่าจะเพิ่มขึ้น

(3) กลางๆ

(4) น่าจะเพิ่มขึ้น

(5) เพิ่มขึ้นอย่างแน่นอน

(97) ฉันไม่ทราบ

(99) ไม่ขอตอบ

7.2 ถ้าหากมีชุดตรวจสำหรับตรวจการติดเชื้อเอชไอวีด้วยตนเอง โดยตรวจจากของเหลวในช่องปาก หรือ ตรวจจากการเจาะเลือด (คือ เก็บของเหลวในช่องปากจากบริเวณเหงือกของท่าน หรือ เจาะเลือดจากปลายนิ้วมือของท่าน และแปลผลการตรวจด้วยตนเอง) โดยชุดตรวจจะมีคำแนะนำเป็นภาษาไทยและมีรูปภาพแสดงวิธีการตรวจ รวมทั้งมีข้อมูลในการติดต่อเจ้าหน้าที่ให้บริการด้านสาธารณสุขเพื่อสนทนาถึงผลตรวจ หรือ กรณีที่ท่านมีคำถามหรือข้อกังวลใดๆ ท่านคิดว่าท่านจะสามารถปฏิบัติตามขั้นตอนที่ระบุด้านล่างด้วยตนเองได้หรือไม่ โปรดให้คะแนนความมั่นใจของท่านจาก 1 ถึง 10 ในแต่ละข้อตามเกณฑ์ที่แสดงดังนี้

	1	2	3	4	5	6	7	8	9	10	
ไม่สามารถทำได้แน่นอน											มั่นใจมากว่าทำได้แน่นอน

7.2.1 ขอชุดตรวจสำหรับตรวจการติดเชื้อเอชไอวีด้วยตนเอง ณ จุดจัดส่ง: _

7.2.2 ปฏิบัติตามวิธีการตรวจที่ระบุในเอกสารให้คำแนะนำ: _

7.2.3 เก็บของเหลวในช่องปาก (ของเหลวบริเวณเหงือก, ไม่ใช่น้ำลาย): _

7.2.4 เก็บหยดเลือดด้วยการเจาะจากปลายนิ้วโดยใช้เข็มเล็กๆ: _

7.2.5 แปลผลการตรวจ: _

7.2.6 ติดต่อเจ้าหน้าที่ให้บริการด้านสาธารณสุขเพื่อสนทนาถึงผลการตรวจ: _

7.2.7 ไปที่คลีนิกเพื่อรับการตรวจการติดเชื้อเอชไอวีอีกครั้งเพื่อยืนยันผล กรณีที่ผลการตรวจด้วยตนเองเป็นบวก: _

7.2.8 ไปรับการรักษาที่คลีนิกที่ให้การดูแลและรักษาผู้ติดเชื้อเอชไอวี ถ้าผลการตรวจการติดเชื้อเอชไอวีของท่านยืนยันว่าเป็นบวก: _

7.3 ท่านเต็มใจจะจ่ายเงิน 400 บาทเพื่อซื้อชุดตรวจสำหรับตรวจการติดเชื้อเอชไอวีด้วยตนเองหรือไม่

(0) ไม่

(1) เต็มใจ

(97) ฉันไม่ทราบ

(99) ไม่ขอตอบ

7.4 ท่านเต็มใจจะจ่ายเงินเพื่อซื้อชุดตรวจสำหรับตรวจการติดเชื้อเอชไอวีด้วยตนเองหรือไม่ ถ้าราคาเพิ่มขึ้นเป็น 500 บาท

(0) ไม่

(1) เต็มใจ

(97) ฉันไม่ทราบ

(99) ไม่ขอตอบ

7.5 สำหรับการซื้อชุดตรวจสำหรับตรวจการติดเชื้อเอชไอวีด้วยตนเอง ท่านยังคงเต็มใจจ่ายเงินซื้อ ถ้า

(1) ราคาลดลงกว่านี้

(2) ราคาเพิ่มมากกว่านี้

(97) ฉันไม่ทราบ

(99) ไม่ขอตอบ

7.6 ราคาสูงสุดเท่าไรที่ท่านจะจ่ายเพื่อซื้อชุดตรวจสำหรับตรวจการติดเชื้อเอชไอวีด้วยตนเอง…………..บาท

7.7 ท่านจะทำอย่างไรถ้าชุดตรวจสำหรับตรวจการติดเชื้อเอชไอวีด้วยตนเองมีราคาสูงเกินไป

(1) จะไม่ทำการตรวจการติดเชื้อเอชไอวี

(2) จะไปรับการตรวจการติดเชื้อเอชไอวีฟรีที่ศูนย์บริการสาธารณสุขของรัฐหรือศูนย์สุขภาพขุมชนที่บริการการตรวจการติดเชื้อเอชไอวีและให้คำปรึกษา

(3) จะไปที่ศูนย์บริการเอกชนและจ่ายค่าตรวจการติดเชื้อเอชไอวีเอง

(97) ฉันไม่ทราบ

(99) ไม่ขอตอบ

7.8 ถ้าท่านต้องการใช้ชุดตรวจสำหรับตรวจการติดเชื้อเอชไอวีด้วยตนเอง ท่านคิดว่า<u>ที่แห่งใดดีที่สุด</u>ในการรับส่งชุดตรวจ (เลือกเพียง 1 คำตอบ)

(1) ศูนย์บริการสุขภาพในชุมชน

(2) ศูนย์บริการสุขภาพของรัฐ

(3) ร้านขายยา

(4) คลีนิกเอกชน

(5) เจ้าหน้าที่ศูนย์บริการสุขภาพในชุมชนขณะออกปฏิบัติหน้าที่

(6) จัดส่งทางไปรษณีย์(ด่วนพิเศษ) หลังจากสั่งทางศูนย์บริการสุขภาพในชุมชน

(7) จัดส่งทางไปรษณีย์(ด่วนพิเศษ) หลังจากสั่งทางอินเตอร์เน็ต

(8) ร้านสะดวกซื้อ เช่น 7-11

(9) ซาวน่า บาร์ หรือร้านนวด

(10) อื่นๆ โปรดระบุ

(97) ฉันไม่ทราบ

(99) ไม่ขอตอบ

7.9 วิธีใดที่ท่านชอบถ้าท่านตัดสินใจจะตรวจการติดเชื้อเอชไอวีด้วยตนเอง (เลือกเพียง 1 คำตอบ)

(1) ใช้ชุดตรวจสำหรับตรวจด้วยตนเอง โดยตรวจจากของเหลวในช่องปาก (ป้ายที่เหงือกเพื่อเก็บของเหลวในช่องปาก)

(2) ใช้ชุดตรวจสำหรับตรวจด้วยตนเอง โดยตรวจจากเลือด (ใช้เข็มเจาะเลือดจากปลายนิ้ว)

(3) ได้ทั้งวิธีตรวจจากของเหลวในช่องปากหรือการเจาะเลือด

(4) ฉันไม่สนใจที่จะตรวจการติดเชื้อเอชไอวีด้วยตนเอง

(97) ฉันไม่ทราบ

(99) ไม่ขอตอบ

ส่วนที่ 8: การใช้สารเสพติด

8.1 ท่านเคยใช้สารเสพติดโดยการฉีด (เพื่อความผ่อนคลายหรือสนุกสนาน โดยไม่ใช่จากการสั่งของแพทย์) หรือไม่

(0) ไม่เคย

(1) เคยใช้ ในช่วง3 เดือนที่ผ่านมา

(2) เคยใช้ ในช่วงระหว่าง 3 เดือนถึง12เดือนก่อนหน้านี้

(3) เคยใช้ เมื่อนานกว่า1ปีมาแล้ว

(99) ไม่ขอตอบ

8.2 ท่านเคยใช้สารเสพติดโดยวิธีการกลืนหรือสูบควันหรือสูดดม(เพื่อความผ่อนคลาย สนุกสนาน โดยไม่ใช่จากการสั่งของแพทย์) หรือไม่

(0) ไม่เคย

(1) เคยใช้ ในช่วง3 เดือนที่ผ่านมา

(2) เคยใช้ ในช่วงระหว่าง 3 เดือนถึง 12 เดือนก่อนหน้านี้

(3) เคยใช้ เมื่อนานกว่า1ปีมาแล้ว

(99) ไม่ขอตอบ

8.3 ถ้าท่านใช้สารเสพติดในช่วง 3 เดือนที่ผ่านมา กรุณาระบุว่าเป็นสารตัวใด โดยใส่หมายเลขในแต่ละข้อ

“0” กรณีที่ไม่ได้ใช้สารนั้นในช่วง 3 เดือนที่ผ่านมา

“1” กรณีที่ใช้สารนั้นในช่วง 3 เดือนที่ผ่านมา

8.3.1 กัญชา __

8.3.2 เมทแอมเฟตามีน (ยาบ้า) __

8.3.3 เอ็กซ์เตรซี่ (ยาอี) __

8.3.4 โคเคน __

8.3.5 เฮโรอีน __

8.3.6 ยาไอซ์ __

8.3.7 บาร์บิตูเรตหรือเบนโซไดอะซีปีน (ยานอนหลับ) __

8.3.8 ดมกาว __

8.3.9 ยาเค (เคตามีน) __

8.3.10 ป๊อปเปอร์ __

8.3.11 อื่นๆ โปรดระบุ…………………………..

8.4 ในช่วง 3 เดือนที่ผ่านมา ท่านเคยใช้ยาเพื่อกระตุ้นความต้องการทางเพศ เช่น ไวอากร้า หรือ ซิอาลีส ก่อนการมีเพศสัมพันธ์หรือไม่

(0) ไม่เคย

(1) เคยใช้

(99) ไม่ขอตอบ

ส่วนที่ 9: การเรียนรู้เพื่อการป้องกัน

9.1 ในช่วง 12 เดือนที่ผ่านมา ท่านเคยเข้าร่วมกิจกรรมบ่อยแค่ไหน (พบตัวต่อตัว หรือ พบเป็นกลุ่มเล็ก) เพื่อสนทนาถึงการป้องกัน, ดูแล และรักษาการติดเชื้อเอชไอวีสำหรับชายที่มีเพศสัมพันธ์กับชาย และ /หรือ สาวประเภทสอง

(0) ไม่เคย

(1) 1 ครั้ง

(2) 2 ครั้ง

(3) 3 ครั้ง

(4) มากกว่า 3 ครั้ง

(97) ฉันไม่ทราบ

(99) ไม่ขอตอบ

9.2 ระหว่างเข้าร่วมกิจกรรมเกี่ยวกับการป้องกันการติดเชื้อเอชไอวีในระยะ 12เดือนที่ผ่านมา ท่านได้รับการส่งต่อเพื่อตรวจการติดเชื้อเอชไอวีหรือไม่

(0) ไม่

(1) ใช่

(99) ไม่ขอตอบ

9.3 กรณีที่ท่านได้รับการส่งต่อเพื่อตรวจการติดเชื้อเอชไอวี ท่านได้รับการตรวจและทราบผลของการตรวจ ใช่หรือไม่

(0) ไม่

(1) ใช่

(2) ไม่ถูกส่งต่อจากการร่วมกิจกรรมดังกล่าว

(99) ไม่ขอตอบ

ส่วนที่ 10: ประวัติการรักษาด้วยยา

10.1 ปัจจุบันนี้ท่านกำลังใช้ยารักษาโรคเรื้อรังอยู่ใช่หรือไม่

(0) ไม่

(1) ใช่

(99) ไม่ขอตอบ

10.2 ในช่วง 3 เดือนที่ผ่านมา ท่านได้ กิน/ฉีดยาฮอร์โมน เพื่อการเปลี่ยนแปลงทางร่างกาย และ/หรือทางอารมณ์ ใช่หรือไม่

(0) ไม่

(1) ใช่

(99) ไม่ขอตอบ

10.3 ท่านเคยรับประทานยาป้องกันการติดเชื้อเอชไอวี (หรือยาเพร็บ) เพื่อลดความเสี่ยงในการติดเชื้อเอชไอวี หรือไม่

(0) ไม่เคย

(1) เคย ในช่วง2เดือนที่ผ่านมา

(2) เคย ระหว่าง 2 ถึง 4 เดือนก่อนหน้านี้

(3) เคย นานกว่า 4 เดือนก่อนหน้านี้

(97) ฉันไม่ทราบหรือไม่เคยได้ยินเรื่องเกี่ยวกับยาเพร็บ

(99) ไม่ขอตอบ

10.4 ท่านเคยรับประทานยา ‘เป๊บ’เพื่อลดความเสี่ยงในการติดเชื้อเอชไอวี หลังจากไปมีเพศสัมพันธ์ที่มีความเสี่ยงต่อการติดเชื้อเอชไอวี เช่น ไม่มีการป้องกันขณะร่วมเพศทางทวารหนักหรือทางช่องคลอด หรือไม่

(0) ไม่เคย

(1) เคย ในช่วง2เดือนที่ผ่านมา

(2) เคย ระหว่าง 2 ถึง 4 เดือนก่อนหน้านี้

(3) เคย นานกว่า 4 เดือนก่อนหน้านี้

(97) ฉันไม่ทราบหรือไม่เคยได้ยินเรื่องเกี่ยวกับยาเป๊บ

(99) ไม่ขอตอบ

คำถามส่วนต่อจากนี้ (ส่วนที่11และส่วนที่12) ใช้สำหรับอาสาสมัครที่เลือกการตรวจการติดเชื้อเอชไอวีด้วยตนเองและตอบแบบสอบถามทางออนไลน์

ส่วนที่ 11: ผลจากการตรวจคัดกรอง

11.1 ผลจากการตรวจคัดกรองเมื่อใช้ ออราควิก/OraQuick คือ

(0) ผลเป็นลบ

(ถ้าเลือกข้อนี้➔จะมีข้อความ: โปรดตรวจอย่างสม่ำเสมอ ถ้าท่านมีโอกาสเสี่ยงต่อการติดเชื้อเอชไอวีควรตรวจอีกครั้งภายใน 3 เดือน หรือ ควรตรวจอีกครั้งภายใน 6 เดือนถ้าท่านไม่มีความเสี่ยงต่อการติดเชื้อเอชไอวี)

(1) ผลเป็นบวก

(ถ้าเลือกข้อนี้➔จะมีข้อความ: ท่านต้องได้รับการตรวจเพิ่มเติมเพื่อยืนยันผลการตรวจการติดเชื้อเอชไอวี โปรดติดต่อศูนย์บริการการตรวจและให้คำปรึกษา ซึ่งระบุในบัตรโครงการของท่าน หรือ ระบุในเว็ปไซต์ของโครงการ)

(2) ผลไม่ชัดเจน

(ถ้าเลือกข้อนี้➔จะมีข้อความ: ท่านต้องได้รับการตรวจเพิ่มเติมเพื่อยืนยันผลการตรวจการติดเชื้อเอชไอวี โปรดติดต่อศูนย์บริการการตรวจและให้คำปรึกษา ซึ่งระบุในบัตรโครงการของท่าน หรือ ระบุในเว็ปไซต์ของโครงการ)

(3) ฉันไม่สามารถแปลผลได้

(ถ้าเลือกข้อนี้➔จะมีข้อความ: ท่านต้องได้รับการตรวจเพิ่มเติมเพื่อยืนยันผลการตรวจการติดเชื้อเอชไอวี โปรดติดต่อศูนย์บริการการตรวจและให้คำปรึกษา ซึ่งระบุในบัตรโครงการของท่าน หรือ ระบุในเว็ปไซต์ของโครงการ)

(99) ไม่ขอตอบ

ส่วนที่ 12: ประสบการณ์ในการใช้ชุดตรวจการติดเชื้อเอชไอวีด้วยตนเอง ออราควิก/OraQuick

12.1 เอกสารให้คำแนะนำวิธีการตรวจการติดเชื้อเอชไอวีด้วยตนเองยากต่อการทำความเข้าใจหรือไม่

(1) ยากมากในการทำความเข้าใจ

(2) ยากพอสมควรในการทำความเข้าใจ

(3) ไม่ยากหรือไม่ง่ายในการทำความเข้าใจ

(4) ค่อนข้างง่ายในการทำความเข้าใจ

(5) ง่ายมากในการทำความเข้าใจ

(97) ฉันไม่ได้อ่านคำแนะนำ

(99) ไม่ขอตอบ

12.2 คำแนะนำวิธีการตรวจการติดเชื้อเอชไอวีด้วยตนเองที่แสดงในวิดีโอ ยากต่อการทำความเข้าใจหรือไม่

(1) ยากมากในการทำความเข้าใจ

(2) ยากพอสมควรในการทำความเข้าใจ

(3) ไม่ยากหรือไม่ง่ายในการทำความเข้าใจ

(4) ค่อนข้างง่ายในการทำความเข้าใจ

(5) ง่ายมากในการทำความเข้าใจ

(97) ฉันไม่ได้ดูวิดีโอให้คำแนะนำ

(99) ไม่ขอตอบ

12.3 การแปลผลการตรวจการติดเชื้อเอชไอวีด้วยตนเองยากหรือไม่

(1) ยากมากในการประเมิน

(2) ยากพอสมควรในการประเมิน

(3) ไม่ยากหรือไม่ง่ายในการประเมิน

(4) ค่อนข้างง่ายในการประเมิน

(5) ง่ายมากในการประเมิน

(99) ไม่ขอตอบ

12.4 วิธีการตรวจการติดเชื้อเอชไอวีด้วยตนเองยากต่อการปฏิบัติหรือไม่

(1) ยากมากในการปฏิบัติ

(2) ยากพอสมควรในการปฏิบัติ

(3) ไม่ยากหรือไม่ง่ายในการปฏิบัติ

(4) ค่อนข้างง่ายในการปฏิบัติ

(5) ง่ายมากในการปฏิบัติ

(99) ไม่ขอตอบ

12.5 ท่านคิดว่าท่านจะแนะนำเพื่อนของท่านให้ใช้การตรวจการติดเชื้อเอชไอวีด้วยตนเองโดยวิธีตรวจจากของเหลวในช่องปากหรือไม่

(1) ไม่แนะนำแน่นอน

(2) อาจจะไม่แนะนำ

(3) เฉยๆ

(4) อาจจะแนะนำ

(5) แนะนำอย่างแน่นอน

(97) ฉันไม่ทราบ

(99) ไม่ขอตอบ
