# Supplementary material for: Uptake of oral fluid-based HIV self-testing among men who have sex with men and transgender women in Thailand
Source: PLoS One. 2021 Aug 16;16(8):e0256094. doi: 10.1371/journal.pone.0256094 (PMC8367007; doi:10.1371/journal.pone.0256094)
Supplement: S1 Codebook — (PDF) [file pone.0256094.s004.pdf]

CODEBOOK FINAL DATASET ORAL FLUID STUDY

Variable Name	Variable Label	Answer Label	Answer Code	Variable Type	Notes	Data collection Tools
**city**	City research site			Numeric		**Enrolment Tools**
		BANGKOK	1			
		CHIANG-MAI	2			
		PATTAYA	3			
**recruit**	Type recruitment - consent done through			Numeric		
		Face-to-Face	1			
		Social Media	2			
**deidentifiedcode**	Code participant deidentified	Open ended		long	This is not an identifier	
**optionhivtest**	HIV test option selected			Numeric	Option 1-3: for partcipnats enrolled under the community approach	
		Assisted HIVST	1		Option 4: only for participants enrolled under the drop-in approach	
		Unassisted HIVST	2			
		Referral to HTC	3			
**deliverykit**	Kit delivery options HIVST only			Numeric		
		Directly Given	1			
		Pick-up Site	2			
		EMS	3			
		N/A	5			
**kp**	Key populations			Numeric		
		MSM	1		men who have sex with men	
		MSW	2		male sex workers	
		TG	3		transgender women	
		TGSW	4		transgender women sex workers	
**msmtg**	MSM vs. TG			Numeric	MSM = MSM + MSW	
		MSM	1		TG = TG + TGSW	
		TG	2			
**sexwork**	Reported sex work in past 3m			Numeric		**Constructed with questions from Questionnnaire**
		No	0			
		Yes	1			
**clientsex**	Reported as a client of sex workers in past 3m			Numeric		
		No	0			
		Yes	1			

Variable Name	Variable Label	Answer Label	Answer Code	Variable Type	Notes	Data collection Tools
**oralresults**	Result OraQuick			Numeric	Result OraQuicK under community and drop-in center approach	**Constructed with CRFs**
		Negative	0			
		Reactive	1			
		Invalid	2			
		Can't be read	3			
		N/A	99			
**bloodresults**	Blood test result_DIC only			Numeric	Result HIV testing under drop-in center approach only	
		Negative	0			
		Positive	1			
		Indeterminate	2			
		Not confirmed yet	97			
		N/A	99			
**linkagehtc**	Status Linkages to HTC			Numeric	If not reactive/invalid/positive==> N/A	
		Linked	1			
		N/A	99			
		LTFU	100			
**statusconfirm**	Status HIV confirmatory test			Numeric	Status at the end of the study - No more "Pending"	
		Pending	0			
		Confirmed	1			
		Known HIV Positive	2			
		N/A	99			
		LTFU	100			
**resultconfirmed**	Result confirmatory test			Numeric	Result of the confirmatory test	
		Negative	0			
		Positive	1			
		Pending	97			
		N/A	99			
		LTFU	100			
**hivstatus**	Final HIV status			Numeric	HIV Status final for all parcipants afer confirmatory HIV testing when it was necessary	
		Negative	0			
		Positive	1			
		Indeterminate	2			
		Not tested yet	96			
		Pending	97			
		LTFU	100			
**typehtcservices**	Type of HTC services			Numeric		
		CBO	1			
		Private	2			
		Governmental	3			
		TRCARC	4			
		Not disclosed	99			
**linkagecst**	Status Linkages to CST			Numeric		
		Linked	1			
		N/A	99			
		LTFU	100			

Variable Name	Variable Label	Answer Label	Answer Code	Variable Type	Notes	Data collection Tools
typecstservices	Type of CST services			Numeric		
		CBO	1			
		Private	2			
		Governmental	3			
		TRCARC	4			
		Not disclosed	99			
LTFU	Loss-to-follow up			Numeric		
		Completed	0			
		LTFU	1			
		N/A	99			
whereltfu	Where LTFU happened			Numeric		
		After Kit dlivery	1			
		Link to HTS services	2			
		Link to CST services	3			
discord	Discordant test results			Numeric		
		No	0			
		Yes	1			
		N/A	99			
evertested	Ever been tested for HIV			Numeric		Constructed with questions in questionnaire
		Never	0			
		Ever	1			
		No answer	99			
firstester	First-time testers			Numeric		
		No	0			
		Yes	1			
		No answer	99			
recentester	HIV test done in past 6m			Numeric		
		No	0			
		Yes	1			
		No answer	99			
frequentester	Repeat testers > = 2 HIV test past 12m			Numeric		
		No	0			
		Yes	1			
		No answer	99			
acceptoral	Acceptability of oral fluid HIVST			Numeric		
		No	0			
		Yes	1			
intentionhivst	Intention for unassisted HIVST in future			Numeric		
		No	0			
		Yes	1			
		No answer	99			

Variable Name	Variable Label	Answer Label	Answer Code	Variable Type	Notes	Data collection Tools
q1age_yr	1.1.1 Reported Age	Open ended		Numeric		
age	reported age	Open ended		Numeric	Use reported age for analysis	
age18	Age groups 18 years			Numeric		
		< 18 years	1			
		≥ 18 years	2			
agecat	Age groups standard			Numeric		
		15-19 years	1			
		20-24 years	2			
		25-29 years	3			
		> 30 years	4			
q1howlong_live	1.10 Living in this city			Numeric		
		visitor	1			
		<1 month	2			
		1–12 months	3			
		>12 months	4			
		No answer	99			
q1sexpartner_12m	1.3 Type partner p12m			Numeric		
		Male	1			
		Female	2			
		Both	3			
		No answer	99			
q1edu	1.4 Highest level educ completed			Numeric		
		No schooling	0			
		Primary	1			
		Secondary	2			
		High-school	3			
		University	4			
		No answer	99			
q1occ	1.5 Current Occupation			Numeric		
		Unemployed	0			
		Student	1			
		Civil servant	2			
		Self-employee	3			
		Employee_not GO	4			
		Other	5			
		No answer	99			

Variable Name	Variable Label	Answer Label	Answer Code	Variable Type	Notes	Data collection Tools
q1married	1.6 Marital status			Numeric		
		Married w woman	1			
		Divorced	2			
		Widowed	3			
		Single	4			
		No answer	99			
q1no_child	1.7 Number of children	Open ended		Numeric		
child	Have children			Numeric		
		No	0			
		Yes	1			
q1livewith	1.8 Living with			Numeric		
		Alone	1			
		W/friends	2			
		W/family	3			
		W/reg. femal. part.	4			
		W/reg. male part.	5			
		No answer	99			
livealone	Living alone			Numeric		
		No	0			
		Yes	1			
		No answer	99			
q1income	1.9 Average monthly income	Open ended		long		
q1income_new	Cleaned_Average monthly income	Open ended		Numeric		
income	Monthly average wage 14,000 THB			Numeric		
		Below	0			
		Above	1			
		No answer	99			
q10nocuredisease	10.1 Taking a long-term Rx for chronic Disease			Numeric		
		No	0			
		Yes	1			
		No answer	99			
q10hormone	10.2 Taken/injected hormones p3m			Numeric		
		No	0			
		Yes	1			
		No answer	99			

Variable Name	Variable Label	Answer Label	Answer Code	Variable Type	Notes	Data collection Tools
**q10prep**	10.3 Ever taken PrEP			Numeric		
		Never	0			
		Past 2 months	1			
		2–4 months	2			
		>4 months	3			
		IDK	97			
		No answer	99			
**q10pep**	10.4 Ever taken PEP			Numeric		
		Never	0			
		Past 2 months	1			
		2–4 months	2			
		>4 months	3			
		IDK	97			
		No answer	99			
**q2condom_fem1**	2.1 Condom Use (CU) last sex w/ fem. reg. part. p3m			Numeric		
		No	0			
		Yes	1			
		N/A	2			
		No answer	99			
**q2condom_fem2**	2.2 CU last sex w/fem. cas./SW part. p3m			Numeric		
		No	0			
		Yes	1			
		N/A	2			
		No answer	99			
**q2condom_male1**	2.3 CU last sex w/ male/TG reg. part. p3m			Numeric		
		No	0			
		Yes	1			
		N/A	2			
		No answer	99			
**q2condom_male2**	2.4 CU last sex w/ male/TG cas/SW/client part. p3m			Numeric		
		No	0			
		Yes	1			
		N/A	2			
		No answer	99			
**q2confreq_male2**	2.5 Freq CU anal sex w/any male/TG part. p3m			Numeric		
		Never	0			
		Sometimes	1			
		Most of the time	2			
		Always	3			
		No anal sex p3m	4			
		No answer	99			

Variable Name	Variable Label	Answer Label	Answer Code	Variable Type	Notes	Data collection Tools
**q2sexrole_male**	2.6 Role anal w/ any male part. in p3m			Numeric		
		Insertive	1			
		Both	2			
		Receptive	3			
		No anal sex p3m	4			
		No answer	99			
**q21stmet_male**	2.7 Place first met last non-reg. male partner			Numeric		
		Social media	1			
		Friend referral	2			
		Private party	3			
		Private sex party	4			
		Bar/discotheque	5			
		Sauna/massage	6			
		open-air	7			
		Other	8			
		No answer	99			
**q2receive_gift**	2.8 Received money/goods for sex p3m			Numeric		
		No	0			
		Yes	1			
		No answer	99			
**q2given_gift**	2.9 Given money/goods for sex p3m			Numeric		
		No	0			
		Yes	1			
		No answer	99			
**q3lasttest**	3.1 When Last HIV test			Numeric		
		Never tested	0			
		< 3 months	1			
		3-6 months	2			
		6–12 months	3			
		>12 months	4			
		No answer	99			
**q3freqtest_12m**	3.2 Freq. HIV test p12m			Numeric		
		No tested p12m	0			
		1 time	1			
		2 times	2			
		3 times	3			
		>3 times	4			
		No answer	99			

Variable Name	Variable Label	Answer Label	Answer Code	Variable Type	Notes	Data collection Tools
q3testplace	3.3 Place of last HIV test			Numeric		
		Never tested	0			
		DIC	1			
		Private	2			
		TRC	3			
		GO hospital	4			
		Mobile services	5			
		research/IBBS	6			
		Other	7			
		No answer	99			
q7pay400b	7.3 WtP 400 THB for HIVST kit			Numeric		
		No	0			
		Yes	1			
		IDK	97			
		No answer	99			
q7pay500b	7.4 WtP medium increase 500 THB for HIVST kit			Numeric		
		No	0			
		Yes	1			
		IDK	97			
		No answer	99			
q7payif	7.5 WtP higher or lower increase for HIVST kit			Numeric		
		Lower increase	1			
		Higher increase	2			
		IDK	97			
		No answer	99			
q7pay_max	7.6 Highest price WtP for HIVST kit	Open ended		Integer		
q7do_what	7.7 If the price HIVST is too high, I will			Numeric		
		No HIV test	1			
		GO/CBO free services	2			
		Private paid services	3			
		IDK	97			
		No answer	99			

Variable Name	Variable Label	Answer Label	Answer Code	Variable Type	Notes	Data collection Tools
q7deliveryplace	7.8 Preferred delivery point for HIVST			Numeric		
		CBO	1			
		GO facility	2			
		Pharmacy	3			
		Private	4			
		CBS	5			
		EMS/CBO	6			
		EMS/Internet	7			
		7/11-type shop	8			
		Sauna/Massage	9			
		Other	10			
		IDK	97			
		No answer	99			
q7likemost	7.9 Preferred type of HIVST			Numeric		
		Oral fluid	1			
		Finger prick	2			
		Any	3			
		not interested HIVST	4			
		IDK	97			
		No answer	99			
druguser	Reported any drug use past 12m			Numeric		
		No	0			
		Yes	1			
		No answer	99			
q8ever_idu	8.1 Ever injected drugs			Numeric		
		Never	0			
		Past 3 months	1			
		3-12 months	2			
		>1 year	3			
		No answer	99			
q8ever_drug	8.2 Ever swallowed/smoked/snorted drugs			Numeric		
		Never	0			
		Past 3 months	1			
		3-12 months	2			
		>1 year	3			
		No answer	99			
q9hivtalk	9.1 Participated HIV prevention activities p12m with CBO			Numeric		
		Never	0			
		1 time	1			
		2 times	2			
		3 times	3			
		> 3 times	4			
		IDK	97			
		No answer	99			

Variable Name	Variable Label	Answer Label	Answer Code	Variable Type	Notes	Data collection Tools
**q12friend_test**	12.5 scale Likely recommend HIVST to friends			Numeric		
		Extremely unlikely	1			
		Unlikely	2			
		Neutral	3			
		Likely	4			
		Extremely likely	5			
		IDK	97			
		No answer	99			
**placemeetmale**	Place met last male _recoded			Numeric		
		Social media	1			
		Private space	2			
		Public space	3			
		Semi-public space	4			
		Other	5			
		No answer	99			
**exposedcbo**	Exposed to CBO past 12m			Numeric		
		No	0			
		Yes	1			
		No answer	99			
**streess**	Reported emotional/cognitive stress after HIVST			Numeric		**Constructed with CRF on AE**
		No	0			
		Yes	1			
**nssi**	Reported non-suicidal self-injury after HIVST			Numeric		
		No	0			
		Yes	1			
**nssi**	Reported non-suicidal self-injury after HIVST			Numeric		
		No	0			
		Yes	1			
**suicideidea**	Reported suicidal ideation after HIVST			Numeric		
		No	0			
		Yes	1			
**suicide**	Reported suicidal attempt after HIVST			Numeric		
		No	0			
		Yes	1			
**binging**	Reported substance binging (drug/alcohol) after HIVST			Numeric		
		No	0			
		Yes	1			
**socailharm**	Reported substance social harm after HIVST			Numeric		
		No	0			
		Yes	1			
**anyae**	Reported at least one adverse event (listed above) after HIVST			Numeric		
		No	0			
		Yes	1			
**multiplrae**	Reported more than one adverse event (listed above) after HIVST			Numeric		
		No	0			
		Yes	1			
